# Human Golgi phosphoprotein 3 is an effector of RAB1A and RAB1B

**DOI:** 10.1371/journal.pone.0237514

**Published:** 2020-08-13

**Authors:** Viviana A. Cavieres, Cristóbal Cerda-Troncoso, Andrés Rivera-Dictter, Rodrigo I. Castro, Charlotte Luchsinger, Natacha Santibañez, Patricia V. Burgos, Gonzalo A. Mardones

**Affiliations:** 1 Department of Physiology, School of Medicine, Universidad Austral de Chile, Valdivia, Chile; 2 Center for Interdisciplinary Studies of the Nervous System (CISNe), Universidad Austral de Chile, Valdivia, Chile; 3 Center for Cell Biology and Biomedicine, School of Medicine and Science, Universidad San Sebastián, Santiago, Chile; 4 Center for Aging and Regeneration (CARE), Facultad de Ciencias Biológicas, Pontificia Universidad Católica de Chile, Santiago, Chile; Institut Jacque Monod, Centre National de la Recherche Scientifique, FRANCE

## Abstract

Golgi phosphoprotein 3 (GOLPH3) is a peripheral membrane protein localized at the *trans*-Golgi network that is also distributed in a large cytosolic pool. GOLPH3 has been involved in several post-Golgi protein trafficking events, but its precise function at the molecular level is not well understood. GOLPH3 is also considered the first oncoprotein of the Golgi apparatus, with important roles in several types of cancer. Yet, it is unknown how GOLPH3 is regulated to achieve its contribution in the mechanisms that lead to tumorigenesis. Binding of GOLPH3 to Golgi membranes depends on its interaction to phosphatidylinositol-4-phosphate. However, an early finding showed that GTP promotes the binding of GOLPH3 to Golgi membranes and vesicles. Nevertheless, it remains largely unknown whether this response is consequence of the function of GTP-dependent regulatory factors, such as proteins of the RAB family of small GTPases. Interestingly, in *Drosophila melanogaster* the ortholog of GOLPH3 interacts with- and behaves as effector of the ortholog of RAB1. However, there is no experimental evidence implicating GOLPH3 as a possible RAB1 effector in mammalian cells. Here, we show that human GOLPH3 interacted directly with either RAB1A or RAB1B, the two isoforms of RAB1 in humans. The interaction was nucleotide dependent and it was favored with GTP-locked active state variants of these GTPases, indicating that human GOLPH3 is a bona fide effector of RAB1A and RAB1B. Moreover, the expression in cultured cells of the GTP-locked variants resulted in less distribution of GOLPH3 in the Golgi apparatus, suggesting an intriguing model of GOLPH3 regulation.

## Introduction

Increasing evidence indicates that defective intracellular membrane trafficking play important roles in tumorigenesis [1]. A distinct putative membrane trafficking regulator is the Golgi-localized protein Golgi phosphoprotein 3 (GOLPH3). GOLPH3 is a highly conserved, peripheral membrane phosphoprotein of ~34 kDa that exchanges dynamically with a large cytosolic pool [2–4]. GOLPH3 is enriched at the *trans*-Golgi network [2], but it is also found on endosomes and the cell surface [4], and its association to Golgi membranes depends on its binding to phosphatidylinositol 4-phosphate (PtdIns4P) [5, 6]. In humans, GOLPH3 is encoded by the chromosomal region 5p13, which is a locus found amplified in many solid tumors [7]. Since this discovery, GOLPH3 has been postulated as the first oncoprotein of the Golgi apparatus, being identified overexpressed in an increasing number of different types of cancer [7–11]. To date, however, it is debated how GOLPH3 acts as an oncoprotein at the Golgi [8, 12–14]. This is mainly due to the multiple cellular activities attributed to GOLPH3. For instance, in addition to initial studies indicating that GOLPH3 is important for Golgi structure and function [2, 4–6, 15], other studies suggest alternative roles, such as the regulation of cell survival after DNA damage [16], the activation of the NF-κB pathway [17], the control of neurogenesis [18], the regulation of the JAK-STAT signaling pathway [19], or an even more puzzling function for a Golgi protein, namely the modulation of mitochondrial homeostasis [20]. As a corollary, many of the tasks of GOLPH3 within cells seem to be consequence of distinct functional states of this protein, such as those that could be promoted by its distinct post-translational modifications or by its distinct dynamic intracellular localizations [2, 4, 21]. Yet, little is known about the precise molecular mechanisms involving GOLPH3 in each of the different cellular processes in which participates, or about the contribution of GOLPH3 to these mechanisms for tumorigenesis.

Similar to other Golgi regulatory proteins, both ATP and GTP promote the association of GOLPH3 to Golgi membranes and vesicles, but by mechanisms that seem to be independent [4]. It is unknown whether this response to nucleotides is consequence of the function of ATP-dependent regulatory proteins, such as protein kinases or phosphoinositide kinases, and/or of GTP-binding regulatory proteins, such as the Golgi-localized members of the RAB, ARF, or ARL families of small GTPases. Nevertheless, recent work shows that the ortholog of GOLPH3 from *Drosophila melanogaster* interacts with the corresponding orthologs of RAB1, RAB5 and RAB11 [22, 23], and that GOLPH3 from the human glioma cell line U87 associates to RAB5 in a protein complex that also contains the epidermal growth factor receptor [24]. Whether this latter association involves a direct interaction between GOLPH3 and RAB5, or if human GOLPH3 interacts directly with any other RAB protein, is unknown. The RAB family of small GTPases is a large group of proteins whose members associate to specific cellular compartments [25]. RABs are key players for membrane trafficking during endocytosis and exocytosis, participating in many specific mechanisms, such as regulating cargo delivery, membrane recycling, maintaining compartment identity, and modulating specialized trafficking functions [26]. In several human cancers it has been found deregulation of several members of the RAB family and their effectors [1]. These GTPases are moderate small monomers within a size range of 20–25 kDa, and they are highly conserved [27]. Similar to other small GTPases, RABs are peripheral membrane proteins functioning as molecular switches, alternating between GTP-bound active and GDP-bound inactive conformations. The structural basis of this capability comprises two regions designated switch I and switch II, which correspond to flexible regions that change conformation depending on binding to GTP or GDP [28]. Consequently, the active conformation of each RAB associates to a specific subcellular compartment for the recruitment of various effectors, orchestrating different cellular processes from these sites [26]. Conversely, the inactive conformation leads to dissociation of RABs from their effectors and their corresponding cognate membrane, and to subsequent recycling of inactive RABs [26]. Here, we tested the hypothesis that human GOLPH3 could be an effector of RAB proteins. We found that GOLPH3 interacted directly with either RAB1A or RAB1B as an effector, but in a fashion that suggests an unexpected model of GOLPH3 regulation.

## Materials and methods

### Recombinant DNA, site-directed mutagenesis and Y2H assay

For the generation of GOLPH3 constructs, a cDNA encoding full-length human GOLPH3 (GenBank/EMBL/DDBJ accession number NM_022130) was acquired from OriGene Technologies (Rockville, MD), and used as a template. Full-length GOLPH3 was obtained by PCR amplification and cloned in-frame into the *Eco*RI and *Sal*I sites of the yeast two-hybrid (Y2H) vector pGBKT7 (Clontech), or into the *Eco*RI and *Sal*I sites of the *E*. *coli* expression vector pGST-Parallel-1 [29]. The pGAD-GH Y2H constructs and the pEGFP mammalian expression constructs encoding either human RAB1A or human RAB1B were a generous gift of S. Miserey-Lenkei (Institut Curie, France). Full-length RAB1A or full-length RAB1B were obtained by PCR amplification and cloned in-frame into the *Eco*RI and *Sal*I sites of vector pGST-Parallel-1. The generation of p53 and T Ag constructs for Y2H were described elsewhere [30]. Single amino acid substitutions were introduced using the QuikChange mutagenesis kit (Stratagene, La Jolla, CA). The nucleotide sequence of all recombinant constructs was confirmed by dideoxy sequencing using the AUSTRAL-*omics* core facility at Universidad Austral de Chile (https://australomics.cl/). Y2H assays were performed as previously described [31].

### Expression and purification of recombinant proteins

Recombinant, untagged GOLPH3, or tagged with an N-terminal glutathione *S*-transferase (GST) followed by a tobacco etch virus (TEV) protease cleavage site were expressed and purified as described previously [21]. Recombinant GST and GST-tagged, wild type or single amino acid substitution variants of human RAB1A or human RAB1B were expressed and purified using a method similar to that for recombinant GOLPH3, with minor modifications. Briefly, expression in *E*. *coli* B834(DE3) (Novagen) was induced with 0.25 mM IPTG at 21°C for 16 h. Pellets of bacteria were resuspended in homogenization buffer (50 mM Tris-HCl, 0.5 M NaCl, 10% glycerol, 5 mM β-mercaptoethanol, and 2 mM phenylmethylsulfonyl fluoride, pH 8.0) and lysed by sonication. After centrifugation at 4°C for 1.5 h, the clarified supernatant was purified on glutathione-Sepharose 4B (GE Healthcare) at 4°C. Alternatively, the GST moiety was removed by His-tagged TEV protease cleavage, and after sequential passage through glutathione-Sepharose 4B and Ni-NTA (QIAGEN) resins, RAB1 proteins were further purified on a Superdex 200 column (GE Healthcare).

### Isothermal titration calorimetry

Purified, recombinant GOLPH3, RAB1A and RAB1B were dialyzed overnight at 4°C against excess isothermal titration calorimetry (ITC) buffer (50 mM Tris-HCl, 150 mM NaCl, 1 mM dithiothreitol (DTT), pH 7.4). ITC measurements were carried out at 25°C using a MicroCal PEAQ-ITC instrument (Malvern Panalytical). Typically, the chamber contained ~0.2 ml of GOLPH3 (100 μM), and either RAB1A or RAB1B (1 mM) added in 18 injections of 3 μl each after a first injection of 0.4 μl. Titration curves were analyzed using MicroCal PEAQ-ITC Analysis Software (Malvern Panalytical). The binding constants and stoichiometry of interactions were calculated by fitting the curves to a one-site model.

### Cell culture, cell transfection and preparation of protein extracts

H4 human neuroglioma cells and HeLa cells were obtained from the American Type Culture Collection, and were maintained in Dulbecco's modified Eagle's medium (DMEM) supplemented with 10% heat-inactivated fetal bovine serum (FBS), 100 U/ml penicillin, 100 μg/ml streptomycin (ThermoFisher), and 5 μg/ml plasmocin (InvivoGen), in a humidified incubator with 5% CO_2_ at 37°C. For transient transfections, cells were either seeded on top of glass coverslips on 24-well plates or directly on 6-well plates. When cells were ~60% confluent, transfections were performed with Lipofectamine 2000 (ThermoFisher), according to the manufacturer's instructions. Preparation of protein extracts from cultured, transfected or non-transfected cells was performed using methods that we have described elsewhere [21].

### GST- and GFP-pulldown assays, protein electrophoresis, immunoblotting and densitometry quantification

For GST-pulldown with recombinant GOLPH3, wild type GST-tagged RAB1A or RAB1B (GST-RAB1A/B) were immobilized on glutathione-Sepharose 4B beads and washed twice in ice-cold binding buffer-1 (20 mM HEPES, 100 mM NaCl, 5 mM MgCl_2_, 1 mM DTT, 1% CHAPS, pH 7.2). Recombinant GOLPH3 was added in increasing concentrations, and the volume adjusted to 500 μl with binding buffer-1. After incubation by end-over-end rotation for 3 h at 4°C, beads were washed four times with 500 μl of binding buffer-1. Bound proteins were eluted with NuPAGE^™^ LDS sample buffer and separated in NuPAGE^™^ 4–12% Bis-Tris gels (ThermoFisher). Proteins in gels were stained with Coomassie Blue G-250 by using SimplyBlue^™^ stain (ThermoFisher). Band intensities of bound GOLPH3 were estimated using ImageJ software (version 1.51s; [32]). Binding constants were obtained by non-linear curve fitting of normalized GOLPH3 band intensities in GraphPad Prism 6 (GraphPad Software). GST-pulldowns were also performed after guanine nucleotide exchange on wild type GST-RAB1A/B. Nucleotide exchange was performed at room temperature (~20°C) as described [33], with some modifications. Briefly, GST-RAB1A/B were immobilized on glutathione-Sepharose 4B beads and washed twice in binding buffer-1. The bound nucleotide was exchanged by washing the beads in binding buffer-1 containing 20 μM of either GDP or Guanosine 5'-[β,γ-imido]triphosphate (GMP-PNP; Sigma-Aldrich) followed by a 60-min incubation in the same buffer containing 1 mM of the corresponding nucleotide. The exchange procedure was repeated twice. Bound nucleotides were stabilized by washing the beads in binding buffer-1 containing 20 μM nucleotide, followed by a 30-min incubation with 1 mM nucleotide in the same buffer. Recombinant GOLPH3 was added at 50 μM adjusting the volume to 500 μl with binding buffer-1 containing 1 mM nucleotide. After incubation by end-over-end rotation for 3 h at 4°C, beads were washed four times with 500 μl of binding buffer-1 containing 1 mM nucleotide. Bound proteins were analyzed as indicated above. As controls, the same nucleotide exchange procedure was performed on GST. For GST-pulldown of endogenous GOLPH3, clarified soluble protein extracts were prepared from H4 cells in ice-cold lysis buffer (20 mM Tris-HCl, 150 mM NaCl, 1 mM DTT, 2% CHAPS, pH 8.0) supplemented with a cocktail of protease inhibitors (416 μM 4-(2-Aminoethyl)benzenesulfonyl fluoride, 0.32 μM Aprotinin, 16 μM Bestatin, 5.6 μM E-64, 8 μM Leupeptin and 6 μM Pepstatin A; Sigma-Aldrich) and a cocktail of phosphatase inhibitors (1 mM NaF, 1 mM Na_3_VO_4_ and 0.3 mM Na_2_P_2_O_7_; Sigma-Aldrich). GST-tagged wild type or single amino acid substitution variants of RAB1A or RAB1B were immobilized on glutathione-Sepharose 4B beads and washed twice in ice-cold binding buffer-1. Soluble protein extracts (0.5 mg) were incubated with immobilized GST or GST-tagged RAB proteins adjusted to a total volume of 1 ml in binding buffer-1 supplemented with the same cocktails of protease and phosphatase inhibitors. After incubation by end-over-end rotation for 3 h at 4°C, beads were washed four times with 500 μl of binding buffer-1. Bound proteins were analyzed by SDS-PAGE and immunoblotting as described [21]. For GFP-pulldown of endogenous GOLPH3, H4 cells were transfected with a plasmid encoding either GFP alone or GFP-tagged versions of either wild type or single amino acid substitution variants of RAB1A or RAB1B. After 16-h, clarified soluble protein extracts were prepared from transfected H4 cells in ice-cold binding buffer-2 (25 mM Tris-HCl, 50 mM NaCl, 0.1% NP-40, pH 7.5) supplemented with the same cocktails of protease and phosphatase inhibitors. GFP-Trap^®^ agarose beads (ChromoTek) were washed with ice-cold binding buffer-2 and incubated with soluble protein extracts (0.2 mg) of transfected cells supplemented with soluble protein extracts (0.3 mg) of non-transfected cells. After incubation by end-over-end rotation for 1.5–4 h at 4°C, GFP-Trap^®^ agarose beads were washed four times with 500 μl of binding buffer-2. Bound proteins were analyzed by SDS-PAGE and immunoblotting as described [21]. The amount of GOLPH3 immunoblot signal from images with unsaturated pixels was estimated using ImageJ software (version 1.51s; [32]).

### Antibodies and cell reagents

We used a mouse monoclonal antibody to β-Actin (clone AC-74; Sigma-Aldrich), a mouse monoclonal antibody to Giantin (clone G1/133; Enzo Life Sciences), a rabbit polyclonal antibody to GOLPH3 (Abcam, cat # ab98023), and a sheep polyclonal antibody to TGN46 (AbD Serotec, cat # AHP500G). We also used a homemade, mouse polyclonal antibody to human GOLPH3 that was described before [34], and a rabbit serum to GFP that was also described elsewhere [35]. HRP–conjugated secondary antibodies were from Jackson ImmunoResearch. The following fluorochrome-conjugated antibodies were from ThermoFisher: Alexa Fluor-594–conjugated donkey anti-rabbit IgG, Alexa Fluor-647–conjugated donkey anti-mouse IgG, Alexa Fluor-350–conjugated donkey anti-sheep IgG and Alexa Fluor-647–conjugated donkey anti-sheep IgG. Primary antibodies were used at a dilution 1/200 to 1/2000. HRP–or Alexa Fluor–conjugated secondary antibodies were used at dilutions 1/1000 to 1/20000, depending on their reactivity. The fluorescent nuclear stain 4’,6-diamidino-2-phenylindole (DAPI) was also from ThermoFisher.

### Fluorescence microscopy and image analysis

For fluorescence microscopy, cells grown in glass coverslips were fixed in 100% methanol or 4% paraformaldehyde and further processed for immunofluorescence as we have described elsewhere [21]. Fluorescence microscopy images were acquired with an AxioObserver.D1 microscope equipped with a PlanApo 63x oil immersion objective (NA 1.4), and an AxioCam MRm digital camera using AxioVision software (Carl Zeiss). We performed quantification of the percentage of fluorescence signal of GOLPH3 associated to Golgi elements by using a method similar to one that we have described elsewhere [31]. Briefly, 12-bit images were acquired under identical settings, avoiding signal saturation and corrected for background, crosstalk and noise signals on each set of images. The resulting processed images obtained from the immunofluorescence using antibodies either to Giantin or to TGN46 were transformed in ImageJ software (version 1.47h; [32]) to binary images after automatic threshold adjusting. Each of the resulting binary images defined a mask that was regarded as the area occupied by Golgi elements. The percentage of localization of GOLPH3 in Golgi elements was calculated for each cell (15 cells from three independent experiments) subtracting the Golgi mask from the total integrated pixel intensity detected in the whole cell of processed images obtained from the immunofluorescence using antibodies to GOLPH3, by using the plugin *Image Calculator* in ImageJ. In the figures this quantification was plotted as percentage of GOLPH3 levels associated to structures decorated with antibodies to Giantin. To evaluate colocalization of fluorescence signals, we obtained the Pearson's correlation coefficient of pairwise comparisons from images acquired and processed as indicated above, using the plugin JACoP [36] implemented in ImageJ. To prepare figures, images were processed with ImageJ software or Adobe Photoshop CS3 software (Adobe Systems, Mountain View, CA).

### Statistical analysis

Statistical analysis was performed using Microsoft Excel for Mac 2011 (Microsoft Corporation). When appropriate, results were represented in graphs depicting the mean ± standard deviation. Statistical significance was determined by two-tailed, paired *t*-test. *P*-values > 0.05 or *≤* 0.05 were regarded as not statistically significant or statistically significant, respectively. In the figures, *P*-values between 0.01 and 0.05 are indicated with one asterisk, *P*-values between 0.001 and 0.01 are indicated with two asterisks, and *P*-values less than 0.001 are indicated with three asterisks.

## Results

### Recombinant GOLPH3 interacts with RAB1A and RAB1B

**Fig 1:**
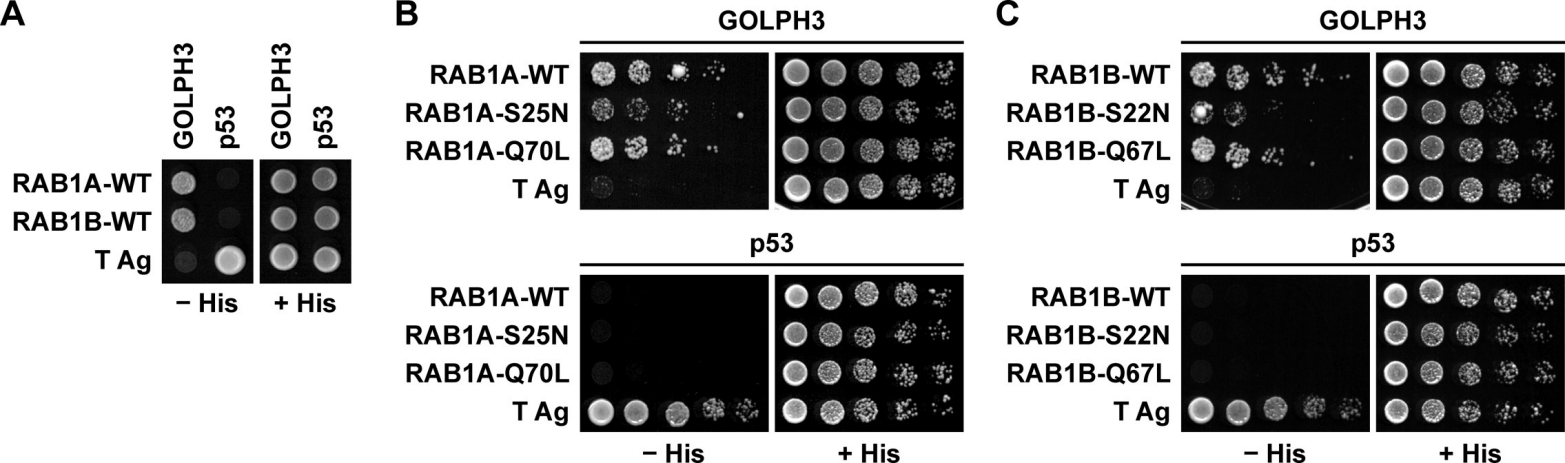
Yeast two-hybrid analysis of the interaction of GOLPH3 with either RAB1A or RAB1B. (A-C) Yeast were co-transformed with plasmids encoding either Gal4bd fused to human GOLPH3 or Gal4ad fused to either RAB1A or RAB1B wild type variants (*RAB1A-WT* and *RAB1B-WT*; A), or to the GDP-locked inactive state variants of either RAB1A or RAB1B (*RAB1A-S25N* and *RAB1B-S22N*; B and C), or to the respective GTP-locked active state variants (*RAB1A-Q70L* and *RAB1B-Q67L*; B and C). Mouse p53 fused to Gal4bd and SV40 large T antigen (T Ag) fused to Gal4ad were used as controls. Co-transformed cells were spotted onto His-deficient (-His) or His-containing (+His) plates and incubated at 30°C. Growth is indicative of interactions. The figure depicts representative images of three independent experiments.

Because GTP promotes the association of GOLPH3 to Golgi membranes and vesicles [4], we tested for direct interaction of human GOLPH3 with various human RAB proteins using the Y2H assay (our unpublished results). We found a robust interaction between GOLPH3 and RAB1A, and also between GOLPH3 and RAB1B (Fig 1A). RAB1A and RAB1B are found at the endoplasmic reticulum-Golgi apparatus interface, and among their functions they regulate vesicular transport between these compartments [37, 38]. Proteins that bind to RABs in a GTP-dependent manner usually interact less efficiently with GDP-locked mutant variants, which remain in the GDP-bound inactive state, and more efficiently with GTP-hydrolysis-deficient variants that remain in the GTP-bound active state [26]. Thus, we compared the interaction of GOLPH3 with the respective wild type, GDP-locked inactive state and GTP-locked active state variants of RAB1A or RAB1B using the Y2H assay. We found that GOLPH3 bound less efficiently to RAB1A-S25N and RAB1B-S22N, the respective GDP-locked inactive state variants (Fig 1B and 1C), as it was expected for a RAB effector. In contrast, the interaction of GOLPH3 with RAB1A-Q70L or RAB1B-Q67L, the respective GTP-locked active state variants, was similar in extent to the interaction of GOLPH3 with the respective wild type variants (Fig 1B and 1C). Nevertheless, together these results suggested that the binding of GOLPH3 to either RAB1A or RAB1B was nucleotide dependent as expected for a RAB effector.

**Fig 2:**
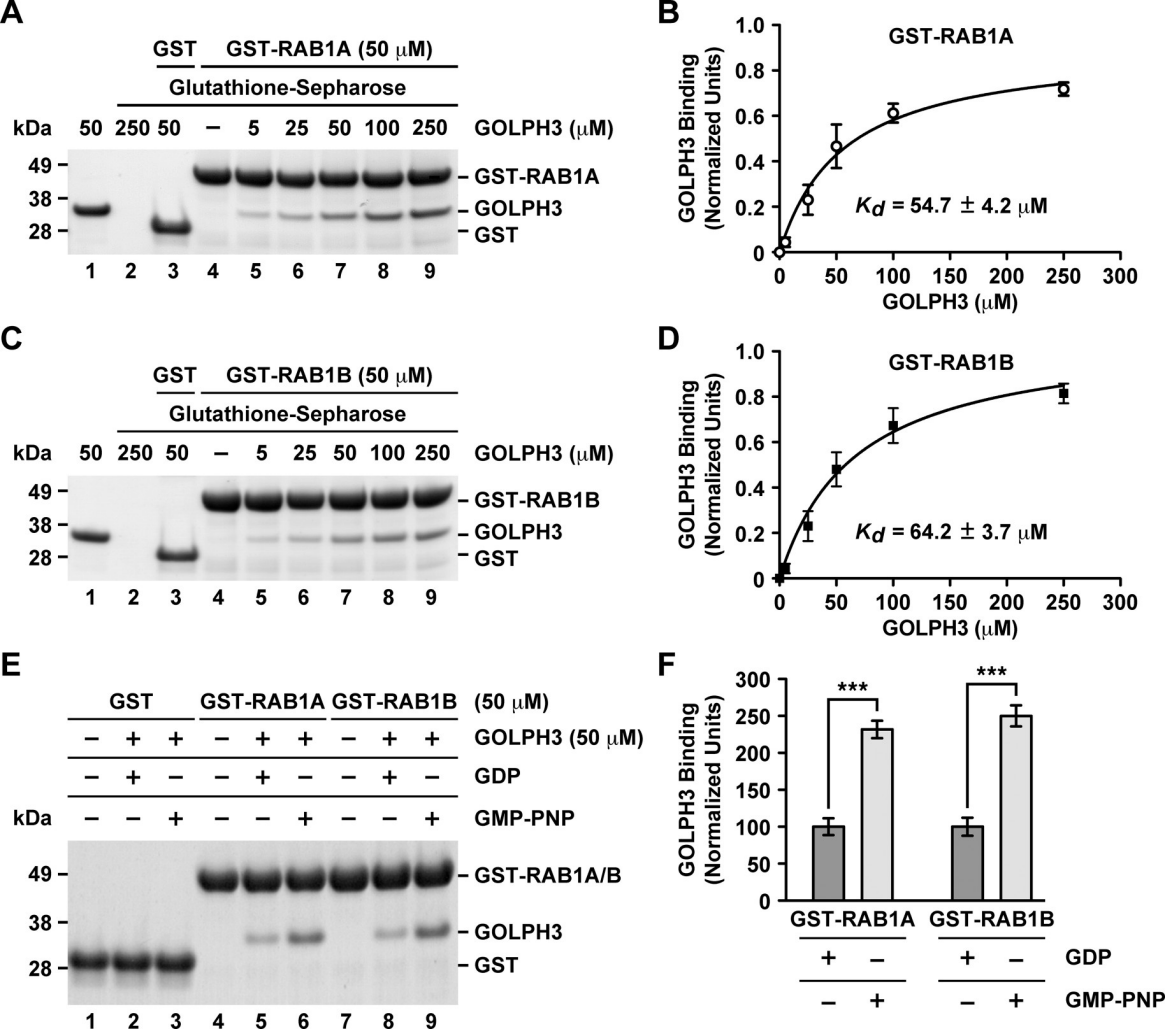
GST-pulldown analysis of the interaction of GOLPH3 with either RAB1A or RAB1B. (A and C) GST-pulldown (A and C, lanes 2–9) using GST-RAB1A at 50 μM (A, lanes 4–9) or GST-RAB1B at 50 μM (C, lanes 4–9) with the indicated different concentrations of GOLPH3 (A and C, lanes 2, 3, 5–9). Lane 1 on each panel was loaded with the indicated amount of GOLPH3 that is equivalent to the amount that was used for the pulldown loaded in lane 7 on each panel. Lanes 2–4 are control pulldowns: lanes 2 are pulldowns using the indicated concentration of GOLPH3 in the absence either of GST-RAB1A (A) or of GST-RAB1B (C); lanes 3 are pulldowns using GST and the indicated concentration of GOLPH3; and lanes 4 are pulldowns using the indicated concentration either of GST-RAB1A (A) or of GST-RAB1B (C) in the absence of GOLPH3. Proteins on glutathione-Sepharose beads were eluted with sample buffer, separated by SDS-PAGE, and stained with Coomassie Blue G-250. The position of the proteins used is indicated on the right. The position of molecular mass markers is indicated on the left. (B and D) Densitometry quantification of the amount of GOLPH3 pulled down as shown in A and C. The amount of GOLPH3 was normalized to the amount in each lane either of GST-RAB1A (B) or of GST-RAB1B (D), and plotted versus each GOLPH3 concentration (n = 3 independent experiments). The data was fitted assuming a 1:1 stoichiometry resulting in the indicated *K*_*D*_. (E) GST-pulldown of GOLPH3 after GDP or GMP-PNP nucleotide exchange on GST-RAB1A (lanes 4–6) or on GST-RAB1B (lanes 7–9). Mock nucleotide exchanges on GST were used as controls (lanes 1–3). (F) Densitometry quantification of the amount of GOLPH3 pulled down as shown in E. The amount of GOLPH3 was normalized to the amount in each lane either of GST-RAB1A (lanes 5 and 6; n = 3 independent experiments) or of GST-RAB1B (lanes 8 and 9; n = 3 independent experiments).

To confirm the interaction of GOLPH3 with RAB1A and RAB1B, we performed GST-pulldown assays followed by SDS-PAGE analysis. GST-RAB1A or GST-RAB1B (wild type versions) were immobilized on glutathione-Sepharose 4B and incubated with increasing amounts of purified recombinant GOLPH3 (Fig 2A and 2C). As control, we incubated GST with GOLPH3 and, as expected, we found little (undetected) or no GOLPH3 pulled down (Fig 2A and 2C, lanes 3). In contrast, GOLPH3 was pulled down by both GST-RAB1A and GST-RAB1B (Fig 2A and 2C, lanes 5–9). Quantification of the amount of bound GOLPH3 indicated a saturation behavior (Fig 2A and 2C, lanes 5–9, and Fig 2B and 2D). Fitting the data assuming a 1:1 stoichiometry between GOLPH3 and either GST-RAB1A or GST-RAB1B resulted in an apparent *K*_*D*_ of 54.7 ± 4.2 μM (Fig 2B) and of 64.2 ± 3.7 μM (Fig 2D), respectively. To analyze whether the interaction of GOLPH3 was nucleotide dependent, we performed nucleotide exchange on either the wild type version of GST-RAB1A or of GST-RAB1B followed by incubation with 50 μM GOLPH3 (a non-saturable concentration; Fig 2B and 2D). We used GDP or GMP-PNP, a non-hydrolysable GTP analog that binds and activate small GTPases [39]. As expected, incubation of GST with GOLPH3 in the presence of either nucleotide resulted in no detection of binding (Fig 2E, lanes 1–3). In contrast, binding of GOLPH3 with either GST-RAB1A or GST-RAB1B was significantly higher in the presence of GMP-PNP (Fig 2, lanes 4–9, and Fig 2F). These results indicate that the binding of GOLPH3 is nucleotide dependent as expected for a RAB effector.

**Fig 3:**
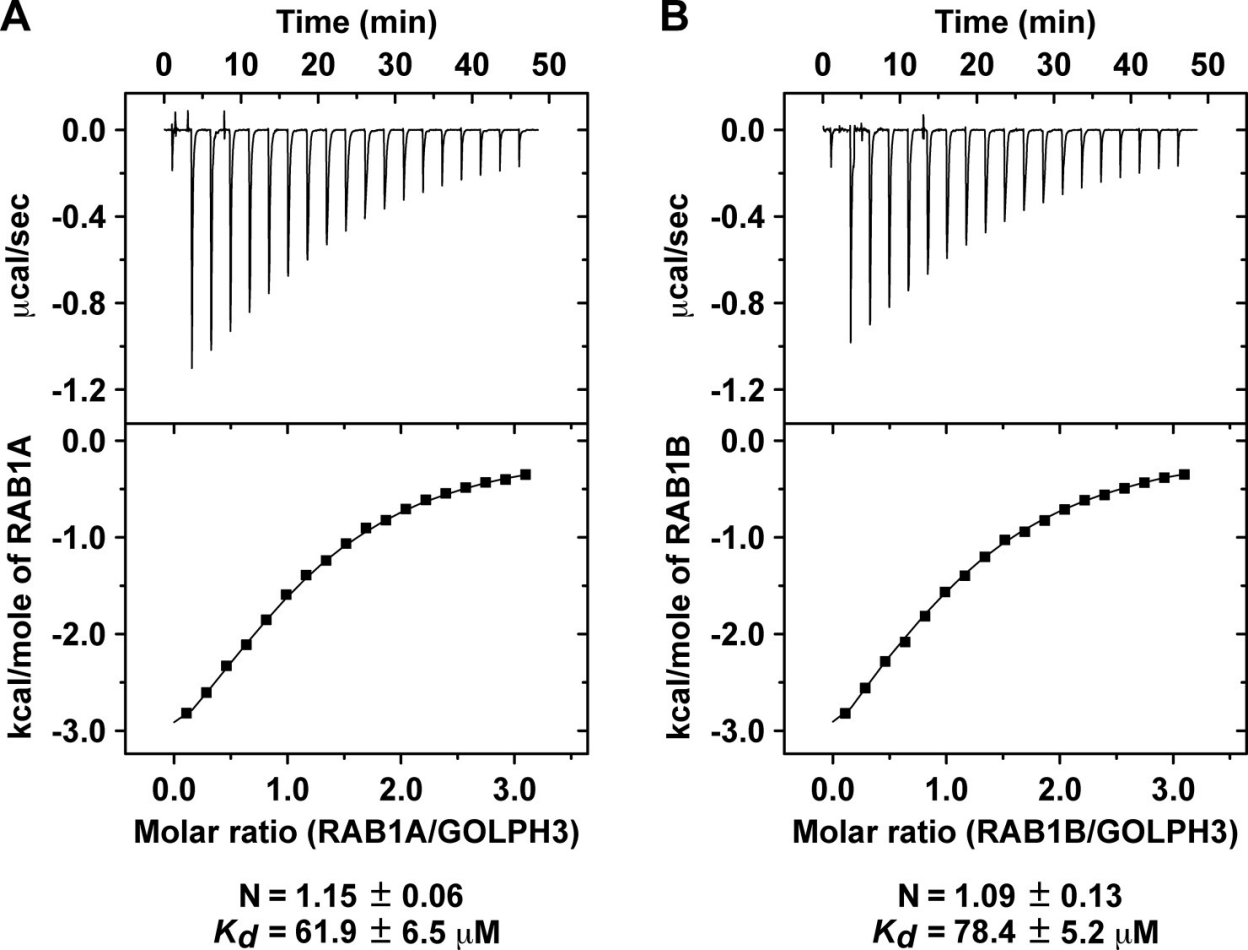
Isothermal titration calorimetry analysis of the interaction of GOLPH3 with either RAB1A or RAB1B. (A-B) Isothermal titration calorimetry of GOLPH3 either with RAB1A (A) or with RAB1B (B). The stoichiometry (N) and *K*_*D*_ for each interaction are expressed as the mean ± SEM (n = 3).

Considering that most RAB effectors show affinities in the low micromolar range [40], the apparent affinities that we found by GST-pulldown seemed weak for a RAB-binding protein. To have additional quantitative assessment of the interactions, we performed isothermal titration calorimetry (ITC) using purified recombinant GOLPH3, RAB1A and RAB1B. Consistent with the GST-pulldown assays, the ITC analysis showed a 1:1 stoichiometry for each interaction, with a *K*_*D*_ of 61.9 ± 6.5 μM for the interaction with RAB1A (Fig 3A) and a *K*_*D*_ of 78.4 ± 5.2 μM for the interaction with RAB1B (Fig 3B). Thus, the ITC data is consistent with the notion of weak, but favorable interactions. However, because we performed ITC without nucleotide exchange on RAB1A or RAB1B, the affinity of GOLPH3 for any of the GTP-bound RAB1 isoforms is likely to be higher. Together, these data support the conclusion that GOLPH3 interacts directly with RAB1A and RAB1B in a nucleotide dependent fashion.

### Endogenous GOLPH3 interacts with RAB1A and RAB1B

**Fig 4:**
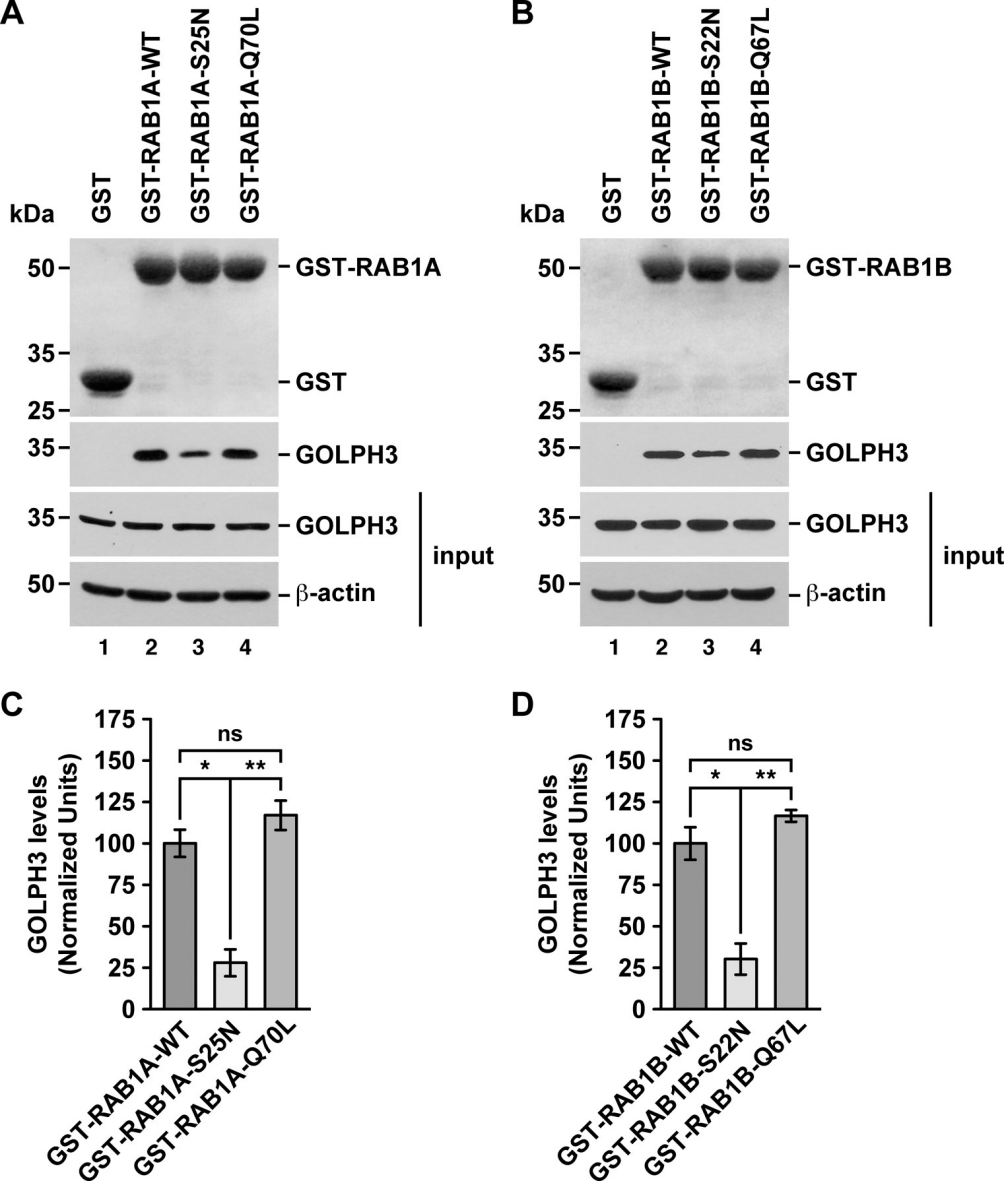
GST-pulldown analysis of the interaction of endogenous GOLPH3 with either RAB1A or RAB1B. (A-B) Pulldowns of the indicated variants of GST-RAB1A (A) or of GST-RAB1B (B) incubated with soluble protein extracts from human neuroglioma H4 cells. Samples of the pulldowns (upper two panels) and of the protein extracts used in the pulldowns (lower two panels; *input*) were processed by SDS-PAGE followed by immunoblotting. Before the immunoblotting of the pulled down samples, nitrocellulose membranes were subjected to Ponceau S staining (upper panels). The position of GST (used as pulldown control) and of the GST-tagged RAB1A or RAB1B variants is indicated on the right. Immunoblottings were carried out using antibodies to detect the proteins indicated on the right of the bottom three panels. The position of molecular mass markers is indicated on the left. (C-D) Densitometry quantification of the amount of GOLPH3 pulled down as shown in the second panel of A and B. The immunoblot signal of anti-β-actin was used as loading control. Bar represents the mean ± standard deviation (n = 3 independent experiments). * *P* < 0.05; ** *P* < 0.01; *ns*, not statistically significant.

To further evaluate whether GOLPH3 interacts with either RAB1A or RAB1B in a nucleotide-dependent manner, we performed GST-pulldown assays using soluble protein extracts from human neuroglioma H4 cells. As expected, GST alone did not pull down endogenous GOLPH3 (Fig 4A and 4B, lanes 1). In agreement with the GST-pulldown using recombinant GOLPH3, GST-RAB1A and GST-RAB1B pulled down endogenous GOLPH3 effectively (Fig 4A and 4B, lanes 2). GST-tagged, GDP-locked inactive state variants of RAB1A and RAB1B, i.e., GST-RAB1A-S25N and GST-RAB1B-S22N, pulled down GOLPH3 significantly less efficiently than the respective wild type variant (Fig 4A and 4B, lanes 2 compared to lanes 3; and 4C and 4D), which is in agreement with the results of the Y2H assay. Pulling down with GST-tagged, GTP-locked active state variants, i.e., GST-RAB1A-Q70L and GST-RAB1B-Q67L, resulted in significantly higher binding to GOLPH3 when compared with the binding of GOLPH3 to the GDP-locked inactive state variants (Fig 4A and 4B, lanes 3 compared to lanes 4; and 4C and 4D). However, when compared with the binding of GOLPH3 to the wild type variants, although the quantification showed increased values (~1.2 times higher for GST-RAB1A-Q70L and ~1.1 times higher for GST-RAB1B-Q67L), the differences were not significant (Fig 4A and 4B, lanes 2 compared to lanes 4; and 4C and 4D). Despite these last results are also in agreement with the results of the Y2H assay, it could be explained by the possibility that the majority of the GST-tagged wild type RAB1A/B variants were bound to GTP provided by the soluble protein extracts in the assay. Thus, together these results also indicate that the binding of GOLPH3 is nucleotide dependent as expected for a RAB effector.

**Fig 5:**
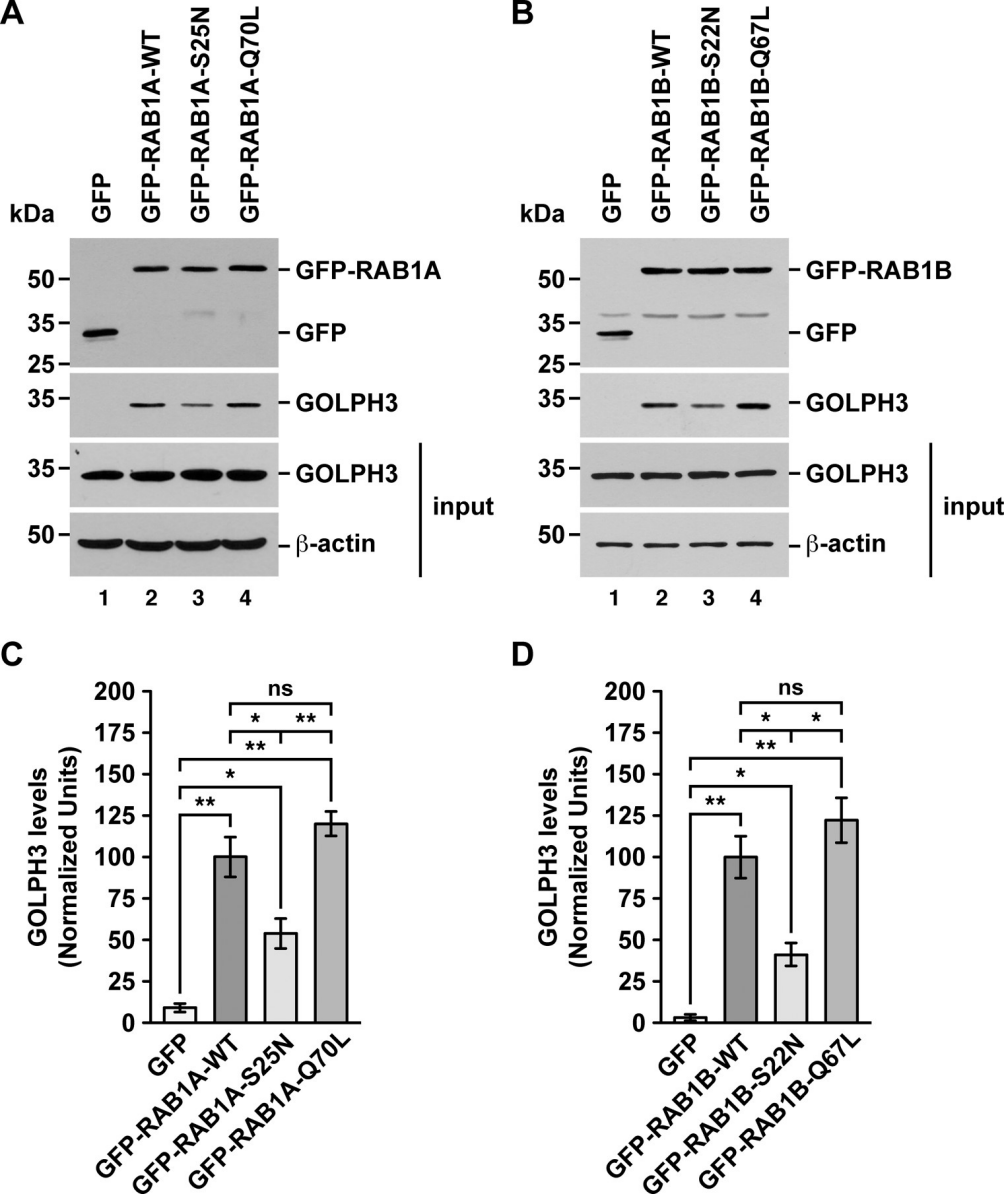
GFP-Trap analysis of the interaction of endogenous GOLPH3 with either RAB1A or RAB1B. (A-B) Soluble protein extracts from human H4 neuroglioma cells transiently expressing the indicated variants of GFP-RAB1A (A) or of GFP-RAB1B (B) were subjected to the GFP-Trap assay. Samples of the GFP traps (upper two panels) and of the protein extracts used in the pulldowns (lower two panels; *input*) were processed by SDS-PAGE followed by immunoblotting. Immunoblottings were carried out using antibodies to detect the proteins indicated on the right. Antibody to GFP was used to detect GFP (used as GFP-Trap control) and the GFP-tagged RAB1A and RAB1B variants. The position of molecular mass markers is indicated on the left. (C-D) Densitometry quantification of the amount of GOLPH3 pulled down as shown in the third panel of A and B. The immunoblot signal of anti-β-actin was used as loading control. Bar represents the mean ± standard deviation (n = 3 independent experiments). * *P* < 0.05; ** *P* < 0.01; *ns*, not statistically significant.

We obtained additional evidence of the distinct binding ability of endogenous GOLPH3 to the different RAB1A and RAB1B variants by performing a GFP-Trap assay also using soluble protein extracts from H4 cells. Although with this assay we found that GFP alone to some extent was able to pull down endogenous GOLPH3 (Fig 5C and 5D), the GFP-tagged wild type variant of both RAB1A and RAB1B pulled down GOLPH3 with significantly higher efficiency (Fig 5A and 5B, lanes 1 compared to lanes 2; and 5C and 5D). Importantly, compared to the binding of endogenous GOLPH3 to the GFP-tagged wild type variants, binding to the GFP-tagged GDP-locked inactive state variants, i.e., GFP-RAB1A-S25N and GFP-RAB1B-S22N, was significantly less efficient (Fig 5A and 5B, lanes 2 compared to lanes 3; and 5C and 5D), in agreement with the GST-pulldown. Likewise, the quantification of the binding of endogenous GOLPH3 to the GFP-tagged GTP-locked active state variants of both RABs, i.e., GFP-RAB1A-Q70L and GFP-RAB1B-Q67L, showed increased values compared to that of the wild type variants (~1.2 times higher for GFP-RAB1A-Q70L and ~1.1 times higher for GFP-RAB1B-Q67L), but both resulted to be not significantly different (Fig 5A and 5B, lanes 2 compared to lanes 4; and 5C and 5D). Nevertheless, similar to the GST-pulldown, the binding of the GFP-tagged GTP-locked state variants was significantly higher to that of the GFP-tagged GDP-locked inactive state variants (Fig 5A and 5B, lanes 3 compared to lanes 4; and 5C and 5D). These results, together with those of the GST-pulldown, confirm that endogenous GOLPH3 interacts with RAB1A and RAB1B, and also confirm the assumption that these interactions are nucleotide dependent as expected for a RAB effector.

### RAB1A and RAB1B affects the distribution of GOLPH3 in cultured cells in an unconventional manner for a RAB effector

**Fig 6:**
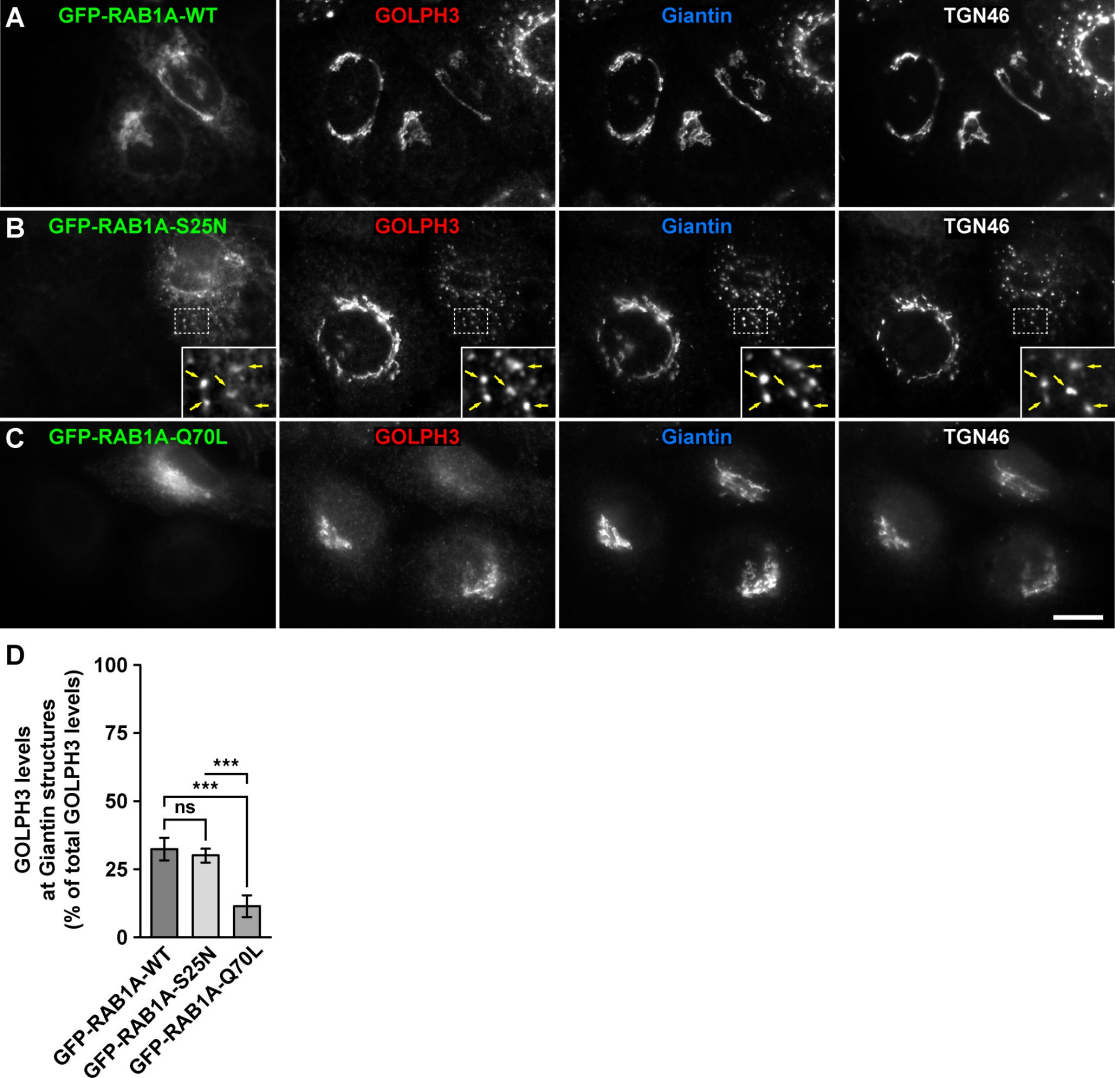
Fluorescence microscopy analysis of the effect of the expression of GFP-tagged RAB1A variants on GOLPH3 subcellular distribution. (A-C) H4 cells grown in glass coverslips were transfected to express either of the indicated GFP-tagged variant of RAB1A (green channels). Cells were fixed, permeabilized, and triple-labeled with rabbit polyclonal antibody to GOLPH3, mouse monoclonal antibody to Giantin and sheep polyclonal antibody to TGN46. Secondary antibodies were Alexa-Fluor-594-conjugated donkey anti-rabbit IgG (red channels), Alexa-Fluor-647-conjugated donkey anti-mouse IgG (blue channels) and Alexa-Fluor-350-conjugated donkey anti-sheep IgG (white channels). Stained cells were examined by fluorescence microscopy. Insets in B: X3 magnification, with arrows indicating colocalization at Golgi punctae. Bar, 10 μm. (D) Quantification as described in *Materials and Methods* of the percentage of fluorescence signal of anti-GOLPH3 associated to Golgi elements decorated with anti-Giantin. Bar represents the mean ± standard deviation (n = 3 independent experiments, and 15 cells in each experiment were analyzed). *** *P* < 0.001; *ns*, not statistically significant.

**Fig 7:**
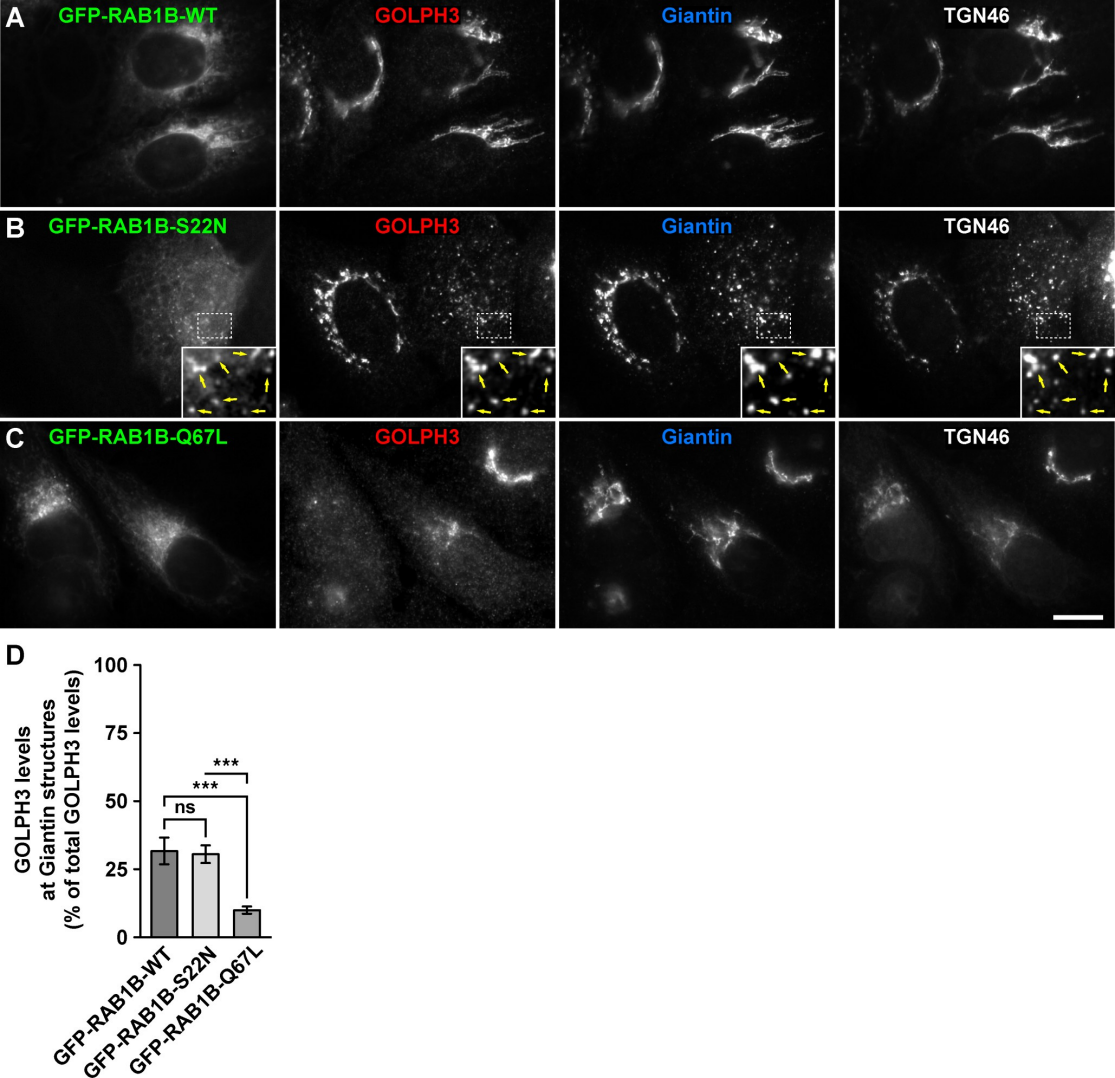
Fluorescence microscopy analysis of the effect of the expression of GFP-tagged RAB1B variants on GOLPH3 subcellular distribution. (A-C) H4 cells grown in glass coverslips were transfected to express either of the indicated GFP-tagged variant of RAB1B (green channels). Cells were fixed, permeabilized, and triple-labeled with rabbit polyclonal antibody to GOLPH3, mouse monoclonal antibody to Giantin and sheep polyclonal antibody to TGN46. Secondary antibodies were Alexa-Fluor-594-conjugated donkey anti-rabbit IgG (red channels), Alexa-Fluor-647-conjugated donkey anti-mouse IgG (blue channels) and Alexa-Fluor-350-conjugated donkey anti-sheep IgG (white channels). Stained cells were examined by fluorescence microscopy. Insets in B: X3 magnification, with arrows indicating colocalization at Golgi punctae. Bar, 10 μm. (D) Quantification as described in *Materials and Methods* of the percentage of fluorescence signal of anti-GOLPH3 associated to Golgi elements decorated with anti-Giantin. Bar represents the mean ± standard deviation (n = 3 independent experiments, and 15 cells in each experiment were analyzed). *** *P* < 0.001; *ns*, not statistically significant.

RAB effectors recruited to intracellular compartments generally have a distinct behavior upon the overexpression of GDP-locked inactive state or GTP-locked active state RAB variants. Overexpression of a GDP-locked inactive RAB usually results in dissociation of cognate effectors from membranes to a cytosolic distribution with dominant-negative effects [41]. Overexpression of GTP-locked active RABs causes instead a tighter association of effectors to membranes, resulting in constitutively active effects [41]. Although our *in vitro* interaction assays indicated a distinct ability of GOLPH3 to interact with RAB1A and RAB1B, it suggested that at least the expression in cultured cells of the GDP-locked inactive state variants should affect GOLPH3 binding to Golgi membranes in a conventional fashion. To test this possibility, we transfected H4 cells with either of the constructs used for the GFP-Trap assay, but after 16-h of expression we processed cells for fluorescence microscopy analysis. We quantified the proportion of GOLPH3 distributed at either the Golgi apparatus or the rest of the cytoplasm by performing triple immunofluorescence detection with antibody to GOLPH3, antibody to Giantin (a *cis*-Golgi protein; [42]) and antibody to TGN46 (a *trans*-Golgi network protein [43]). As expected, compared to GFP-expressing cells, the expression of GFP-RAB1A or of GFP-RAB1B, the respective GFP-tagged wild type RAB1A and RAB1B variants, which localize mostly at the endoplasmic reticulum and the Golgi apparatus [37, 38], did not affect GOLPH3 distribution (i.e., Golgi-associated versus in the rest of the cytoplasm), as indicated by colocalization at the perinuclear region with both Giantin (Pearson's correlation coefficient [*r*] of 0.931 ± 0.030 in GFP-expressing cells versus 0.926 ± 0.045 in GFP-RAB1A-expressing cells [p>0.05], and 0.933 ± 0.052 in GFP-RAB1B-expressing cells [p>0.05]; n = 15 cells analyzed for each comparison; Figs 6A and 7A, and S1 and S2 Figs) and TGN46 (*r* = 0.940 ± 0.015 in GFP-expressing cells versus 0.932 ± 0.033 in GFP-RAB1A-expressing cells [p>0.05], and 0.915 ± 0.042 in GFP-RAB1B-expressing cells [p>0.05]; n = 15 cells analyzed for each comparison; Figs 6A and 7A, and S1 and S2 Figs) of similar levels of GOLPH3-associated fluorescence (Figs 6D and 7D, and S3C and S4C Figs). Expression of the GDP-locked inactive state variants of RAB1A or of RAB1B results in dispersal of Golgi elements and cytosolic distribution of some of their effectors, such as the proteins p115 and GM130 [41, 44]. We found that expression of either of the GFP-tagged, GDP-locked inactive state variants (GFP-RAB1A-S25N or GFP-RAB1B-S22N) resulted in dispersal of the Golgi apparatus, as indicated by colocalization of Giantin and TGN46 in scattered punctae (*r* = 0.894 ± 0.030 in GFP-RAB1A-expressing cells versus 0.896 ± 0.039 in GFP-RAB1A-S25N-expressing cells [p>0.05], and 0.901 ± 0.025 in GFP-RAB1B-expressing cells versus 0.877 ± 0.040 in GFP-RAB1B-S22N-expressing cells [p>0.05]; n = 15 cells analyzed for each comparison; Figs 6B and 7B). Unexpectedly, expression of either of the GDP-locked variants did not affect the distribution of GOLPH3, but instead the proportion of GOLPH3-associated fluorescence at Golgi punctae (30.1 ± 2.6% in GFP-RAB1A-S25N-expressing cells, Fig 6B and 6D, and 30.5 ± 3.2% in GFP-RAB1B-S22N-expressing cells, Fig 7B and 7D) was similar to that associated to the Golgi apparatus of cells expressing either of the respective wild type variant (32.5 ± 4.2% in GFP-RAB1A-expressing cells, Fig 6B and 6D, and 31.7 ± 4.9% in GFP-RAB1B-expressing cells, Fig 7B and 7D). In contrast to the expression of the GDP-locked variants, expression of the GFP-tagged, GTP-locked active state variants (GFP-RAB1A-Q70L or GFP-RAB1B-Q67L) resulted in no dispersal of the Golgi apparatus, as indicated by colocalization of Giantin and TGN46 at the perinuclear region (*r* = 0.894 ± 0.030 in GFP-RAB1A-expressing cells versus 0.896 ± 0.025 in GFP-RAB1A-Q70L-expressing cells [p>0.05], and 0.901 ± 0.025 in GFP-RAB1B-expressing cells versus 0.895 ± 0.054 in GFP-RAB1B-Q67L-expressing cells [p>0.05]; n = 15 cells analyzed for each comparison; Figs 6C and 7C). Strikingly to us, expression of either of the GTP-locked variants resulted in a significant decrease of the proportion of GOLPH3-associated fluorescence at the Golgi (11.5 ± 4.0% in GFP-RAB1A-Q70L-expressing cells, Fig 6C and 6D, and 10.0 ± 1.4% in GFP-RAB1B-Q67L-expressing cells, Fig 7C and 7D) compared to cells expressing either the respective wild type variant or the respective GDP-locked variant (Figs 6D and 7D). Together, these results strongly suggest that RAB1A and RAB1B regulate the subcellular distribution of GOLPH3 in an unusual manner.

## Discussion

In our present work, we provide evidence demonstrating that human GOLPH3 interacts directly either with RAB1A or with RAB1B, in agreement with previous findings indicating that Golph3 in *Drosophila melanogaster* is an effector of Rab1 [23]. This last conclusion is supported by the fact that compared to the interaction with wild type Rab1, Golph3 interacts with higher avidity with the GTP-locked active variant of Rab1 and with lower avidity with the corresponding GDP-locked inactive variant [23]. This dependency on the state of RABs nucleotide loading for interaction is a hallmark of RABs effectors binding [26]. In contrast, our Y2H analysis, as well as our GST-pulldown and GFP-Trap analyses using endogenous GOLPH3, showed that although GOLPH3 interacted with less avidity with the GDP-locked variants of RAB1A and RAB1B, the interaction with the corresponding GTP-locked variants showed to be similar to that with the wild type forms. Nevertheless, our pulldowns after nucleotide exchange on either wild type GST-RAB1A or wild type GST-RAB1B showed that recombinant GOLPH3 bound in a nucleotide-dependent manner. This could be explained if in both the Y2H assay and the pulldown experiments, using soluble protein extracts, the wild type variants of RAB1A and RAB1B are mostly GTP bound. Alternatively, our experimental setup might have not considered some biochemical requirement that could facilitate higher avidity of GOLPH3 for the GTP-locked variants of RAB1A and RAB1B. In this regard, different types of cells contain distinct phosphorylation pools of GOLPH3, with less-phosphorylated forms enriched in the cytosol and more-phosphorylated forms associated to Golgi membranes [2, 21]. Because our biochemical analyses were performed with cell lysates prepared in the presence of phosphatase inhibitors, the most likely GOLPH3 forms present in our assays were those more phosphorylated, which might have a suboptimal avidity for RAB1A and RAB1B. Such a dependency on the state of phosphorylation of a RAB protein effector has been reported for the budding yeast *Saccharomyces cerevisiae*. Phosphorylation of the HOPS complex, which is an effector of the RAB7 ortholog Ypt7, is necessary for the vacuole membrane fusion promoted by the GTP-bound active state of Ypt7 [45]. The clarification whether the state of phosphorylation affects the binding of GOLPH3 to RAB1A and RAB1B will need further investigation.

An interesting observation is that in *Drosophila melanogaster* Golph3 interacts also with Rab5 and Rab11 [22], behaving as canonical effector of at least Rab11 [23]. Moreover, in human U87 glioma cells, GOLPH3 is bound to a complex that contains RAB5 and the epidermal growth factor receptor [24], although a direct interaction between GOLPH3 and RAB5 remains to be established. The long lasting model of RABs function indicates that each RAB recruits a set of specific effectors, and that each effector performs a specific and distinct function [46]. However, some effectors interact and are regulated by two or more RABs. For instance, Rabenosyn-5, a protein initially identified as RAB5 effector, also binds to RAB4, functioning as divalent RAB effector for the regulation of sub-compartmental organization and sorting in endosomes [47]. Likewise, the RAB11 effector RCP is also effector of RAB4 and RAB14, but in different fashions [48–50]. Thus, the interaction of Golph3 with multiple Rabs in *Drosophila melanogaster* suggests that GOLPH3 in humans could also be an effector of multiple RABs. This possibility could explain how under certain conditions GOLPH3 associates to subcellular compartments other than the Golgi apparatus, including to endosomes [4, 7]. RAB1A and RAB1B localize in the endoplasmic reticulum-Golgi apparatus interface [37, 38]. In contrast, RAB5 localizes in endosomes, consistent with the recruitment of GOLPH3 at these sites in several types of cells under some conditions [4, 7, 24]. Binding of GOLPH3 to PtdIns4P seems to be sufficient for GOLPH3 recruitment to Golgi membranes [5, 6], suggesting that the interaction with RAB1A and RAB1B could be instead important for the regulation of downstream functions. Although PtdIns4P is enriched in Golgi membranes, it is also found in other compartments such as the cell surface and endosomes [51]. Therefore, GOLPH3 could be recruited to endosomes also in a PtdIns4P-dependent fashion where its binding to RAB5 could also be important for its regulation. Similarly, in *Drosophila melanogaster* the interaction of Golph3 with Rab5 and Rab11 could be important for the regulation of Golph3 function in endosomes. Remarkably, the affinity of interaction of recombinant Golph3 and Rab11 is 180 nM [22], indicating a tight functional association in endosomes. In contrast, in our *in vitro* analyses we observed that the associations between human GOLPH3 and either RAB1A or RAB1B were in the order of 50–80 μM, interactions of rather lower affinity. Thus, these *K*_*D*_ values were in a range typical of weak transient interactions, which nevertheless are important and abundant in biological systems (e.g. [52]). This suggests at least two not mutually exclusive possibilities. First, that at the Golgi apparatus such a tight interaction might not be necessary for a functional association, and second, that in human cells a more optimal interaction might require one or more elements not considered in our *in vitro* assays with recombinant GOLPH3, RAB1A and RAB1B, such as a functional phosphorylation. More analyzes will be necessary to distinguish between these possibilities.

An unexpected result was that the expression of either GDP-locked or GTP-locked variants of RAB1A or RAB1B resulted in subcellular distributions of GOLPH3 opposite to those likely for canonical effectors, i.e., GOLPH3 remained associated to dispersed Golgi punctae of cells expressing the GDP-locked RABs variants, and less associated to the Golgi in cells expressing GTP-locked RABs variants. A possible explanation to these outcomes is that the association of GOLPH3 to the Golgi apparatus does not depend on the binding to RAB1A or RAB1B, consistent with the finding that it depends on its binding to PtdIns4P [5, 6]. Instead, binding to any of the RAB1 isoforms could proceed for two mechanistically separated actions: an initial nucleotide-semi independent binding for the regulation of GOLPH3 function at the Golgi apparatus, followed by a nucleotide-dependent interaction necessary for subsequent GOLPH3 recycling to the cytosol. This last mechanism could progress by either of the RAB1 isoform directly promoting on GOLPH3 its dissociation from Golgi membranes, or by assisting its dissociation promoted by an additional recruited effector. Although at this point this is an entirely speculative model, tripartite complexes formed by RAB proteins have been described. For instance, for the transport of cholesterol at the interface between endosomes and the endoplasmic reticulum, GTP-locked RAB7 interacts with its effector RILP using its inter-switch region, and to the oxysterol-binding protein ORP1 in a mode that is independent of nucleotide binding and by using a distinct site [53].

During the last ten years, increasing experimental evidence positions GOLPH3 as an important player in cancer biology [8, 12, 13, 54]. It is well established that the increase in the levels of GOLPH3 promotes tumorigenic phenotypes in different types of cells, indicating that it is a true oncoprotein [7, 8]. Different cell processes implicated in tumorigenesis are enhanced by GOLPH3, including cell proliferation, cell migration and cell invasion [7, 34, 55–58]. Moreover, GOLPH3 is implicated in a variety of diverse functions that may or not contribute to malignant transformation, such as cell survival after DNA damage [16], maintenance of Golgi apparatus structure [5], glycosyltransferases localization [59–61], or modulation of mitochondrial activity [20]. However, it is not yet well understood how GOLPH3 functions at the molecular level in these processes, and how it is regulated to perform these functions. Importantly, GOLPH3 drives a PtdIns4P-dependent, Golgi apparatus membrane curvature and tubulation for the efficient formation of anterograde membrane trafficking intermediates that eventually reach the cell surface [4, 21, 62]. An intriguing possibility is that RAB1A and RAB1B regulate GOLPH3 to lead this mechanism at the Golgi apparatus, which could be necessary for some of the diverse tumorigenic functions attributed to GOLPH3. Interestingly, the overexpression of the Golgi phosphatidylinositol transfer protein PITPNC1 in breast cancer cell lines promotes the formation of a protein complex with RAB1B, which subsequently seems to recruit GOLPH3 to the Golgi, resulting in enhanced secretion of pro-invasive and pro-metastatic mediators [63]. However, a direct interaction between mammalian GOLPH3 and RAB1B (or of GOLPH3 and the PITPNC1/RAB1B complex) was not reported. It will be important to determine whether the recruitment of GOLPH3 to Golgi membranes in cells overexpressing PITPNC1 is part of a distinct RAB1B-regulated mechanism. RAB1A, on the other hand, has not yet been implicated in any mechanism involving GOLPH3. Nevertheless, the lack of selectivity of GOLPH3 for the binding to any of the RAB1 isoforms could be explained by functional redundancy. In any case, RAB1A has been implicated in cancer progression and poor prognosis [64]. Moreover, because increased expression of either RAB1A or RAB1B has been found in several types of cancer, including prostate cancer [65], hepatocellular carcinoma [66–68] and colorectal carcinoma [69, 70], it would be not surprising if their effects are synergistic with that of overexpressed GOLPH3. Further studies addressing the functional role of GOLPH3 binding to RAB1A and RAB1B will provide insights on GOLPH3 function in normal and pathological conditions such as in cancer.

## Supporting information

S1 Fig Merged images of the fluorescence microscopy analysis shown in Fig 6. Cells were triple-labeled with rabbit polyclonal antibody to GOLPH3, mouse monoclonal antibody to Giantin and sheep polyclonal antibody to TGN46. Secondary antibodies were Alexa-Fluor-594-conjugated donkey anti-rabbit IgG, Alexa-Fluor-647-conjugated donkey anti-mouse IgG and Alexa-Fluor-350-conjugated donkey anti-sheep IgG. Stained cells were examined by fluorescence microscopy. Insets in B: X3 magnification, with arrows indicating colocalization at Golgi punctae. Bar, 10 μm. For comparison of the fluorescence signals, pairs of images were pseudocolored as indicated.
(TIF)

S2 Fig Merged images of the fluorescence microscopy analysis shown in Fig 7. Cells were triple-labeled with rabbit polyclonal antibody to GOLPH3, mouse monoclonal antibody to Giantin and sheep polyclonal antibody to TGN46. Secondary antibodies were Alexa-Fluor-594-conjugated donkey anti-rabbit IgG, Alexa-Fluor-647-conjugated donkey anti-mouse IgG and Alexa-Fluor-350-conjugated donkey anti-sheep IgG. Stained cells were examined by fluorescence microscopy. Insets in B: X3 magnification, with arrows indicating colocalization at Golgi punctae. Bar, 10 μm. For comparison of the fluorescence signals, pairs of images were pseudocolored as indicated.
(TIF)

S3 Fig Fluorescence microscopy analysis of the effect of the expression of GFP or wild type GFP-RAB1A on GOLPH3 subcellular distribution. H4 cells grown in glass coverslips were transfected to express GFP (used as control; A, green channel), or the wild type GFP-tagged variant of RAB1A (B, green channel). Cells were fixed, permeabilized, and double-labeled with rabbit polyclonal antibody to GOLPH3 and sheep polyclonal antibody to TGN46. Secondary antibodies were Alexa-594-conjugated donkey anti-rabbit IgG (red channels) and Alexa-647-conjugated donkey anti-sheep IgG (blue channels). Nuclei were stained with DAPI (gray channels). Stained cells were examined by fluorescence microscopy. Merging green, red, blue and grey channels generated the fourth image on each row; yellow indicates overlapping localization of the red and green channels, cyan indicates overlapping localization of the green and blue channels, magenta indicates overlapping localization of the red and blue channels, and white indicates overlapping localization of all three channels. Bar, 10 μm. (C) Quantification as described in *Materials and Methods* of the percentage of fluorescence signal of anti-GOLPH3 associated to Golgi elements decorated with anti-TGN46. Bar represents the mean ± standard deviation (n = 3 independent experiments, and 15 cells in each experiment were analyzed); *ns*, not statistically significant.
(TIF)

S4 Fig Fluorescence microscopy analysis of the effect of the expression of GFP or wild type GFP-RAB1B on GOLPH3 subcellular distribution. H4 cells grown in glass coverslips were transfected to express GFP (used as control; A, green channel), or the wild type GFP-tagged variant of RAB1B (B, green channel). Cells were fixed, permeabilized, and double-labeled with rabbit polyclonal antibody to GOLPH3 and sheep polyclonal antibody to TGN46. Secondary antibodies were Alexa-594-conjugated donkey anti-rabbit IgG (red channels) and Alexa-647-conjugated donkey anti-sheep IgG (blue channels). Nuclei were stained with DAPI (gray channels). Stained cells were examined by fluorescence microscopy. Merging green, red, blue and grey channels generated the fourth image on each row; yellow indicates overlapping localization of the red and green channels, cyan indicates overlapping localization of the green and blue channels, magenta indicates overlapping localization of the red and blue channels, and white indicates overlapping localization of all three channels. Bar, 10 μm. (C) Quantification as described in *Materials and Methods* of the percentage of fluorescence signal of anti-GOLPH3 associated to Golgi elements decorated with anti-TGN46. Bar represents the mean ± standard deviation (n = 3 independent experiments, and 15 cells in each experiment were analyzed); *ns*, not statistically significant.
(TIF)

S1 Raw images (PDF)
