# Supplementary figures and images for: Human Golgi phosphoprotein 3 is an effector of RAB1A and RAB1B
Source: PLoS One. 2020 Aug 13;15(8):e0237514. doi: 10.1371/journal.pone.0237514 (PMC7425898; doi:10.1371/journal.pone.0237514)

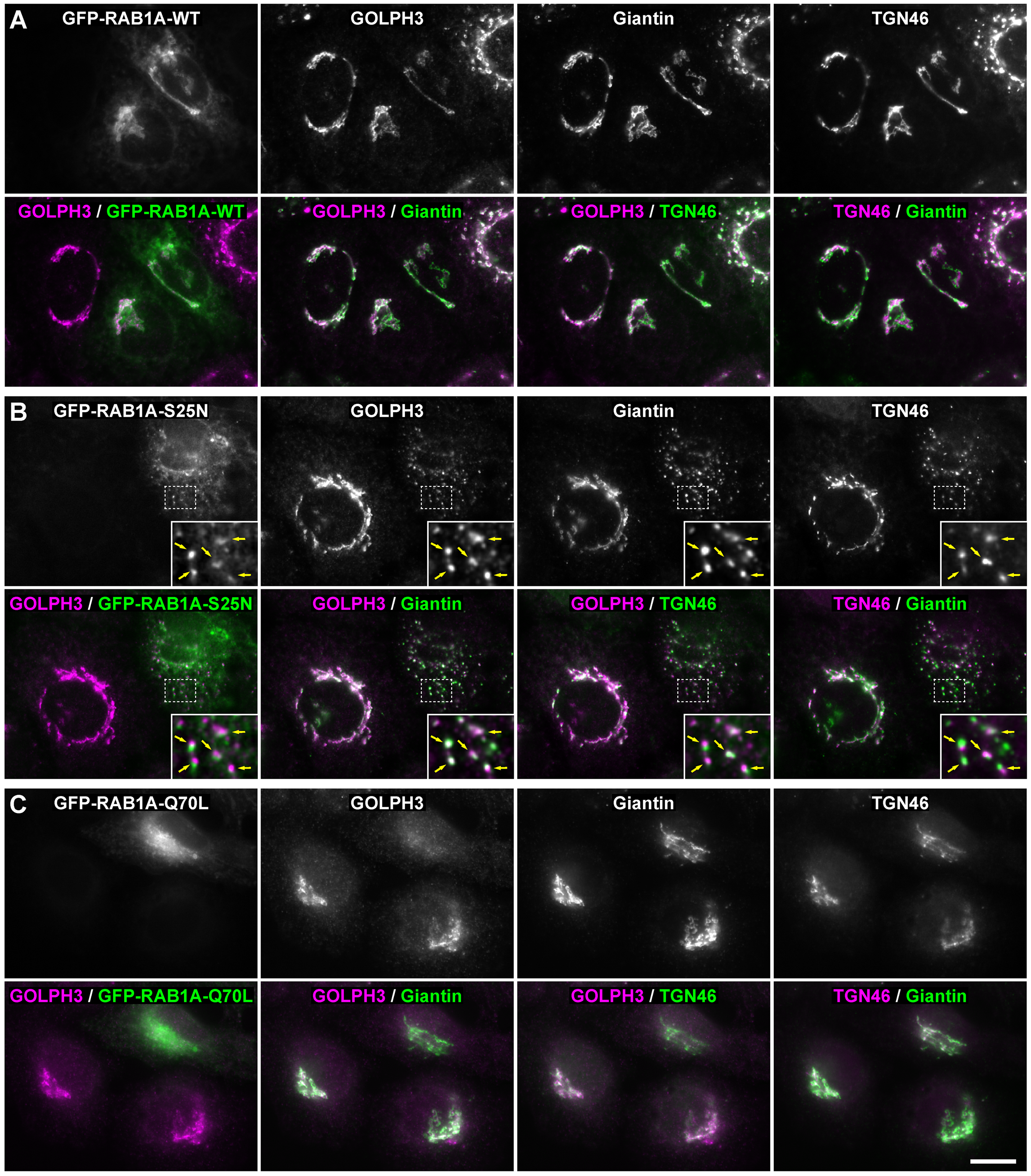

Supplement: S1 Fig — Cells were triple-labeled with rabbit polyclonal antibody to GOLPH3, mouse monoclonal antibody to Giantin and sheep polyclonal antibody to TGN46. Secondary antibodies were Alexa-Fluor-594-conjugated donkey anti-rabbit IgG, Alexa-Fluor-647-conjugated donkey anti-mouse IgG and Alexa-Fluor-350-conjugated donkey anti-sheep IgG. Stained cells were examined by fluorescence microscopy. Insets in B: X3 magnification, with arrows indicating colocalization at Golgi punctae. Bar, 10 μm. For comparison of the fluorescence signals, pairs of images were pseudocolored as indicated. (TIF) [file pone.0237514.s001.tif]

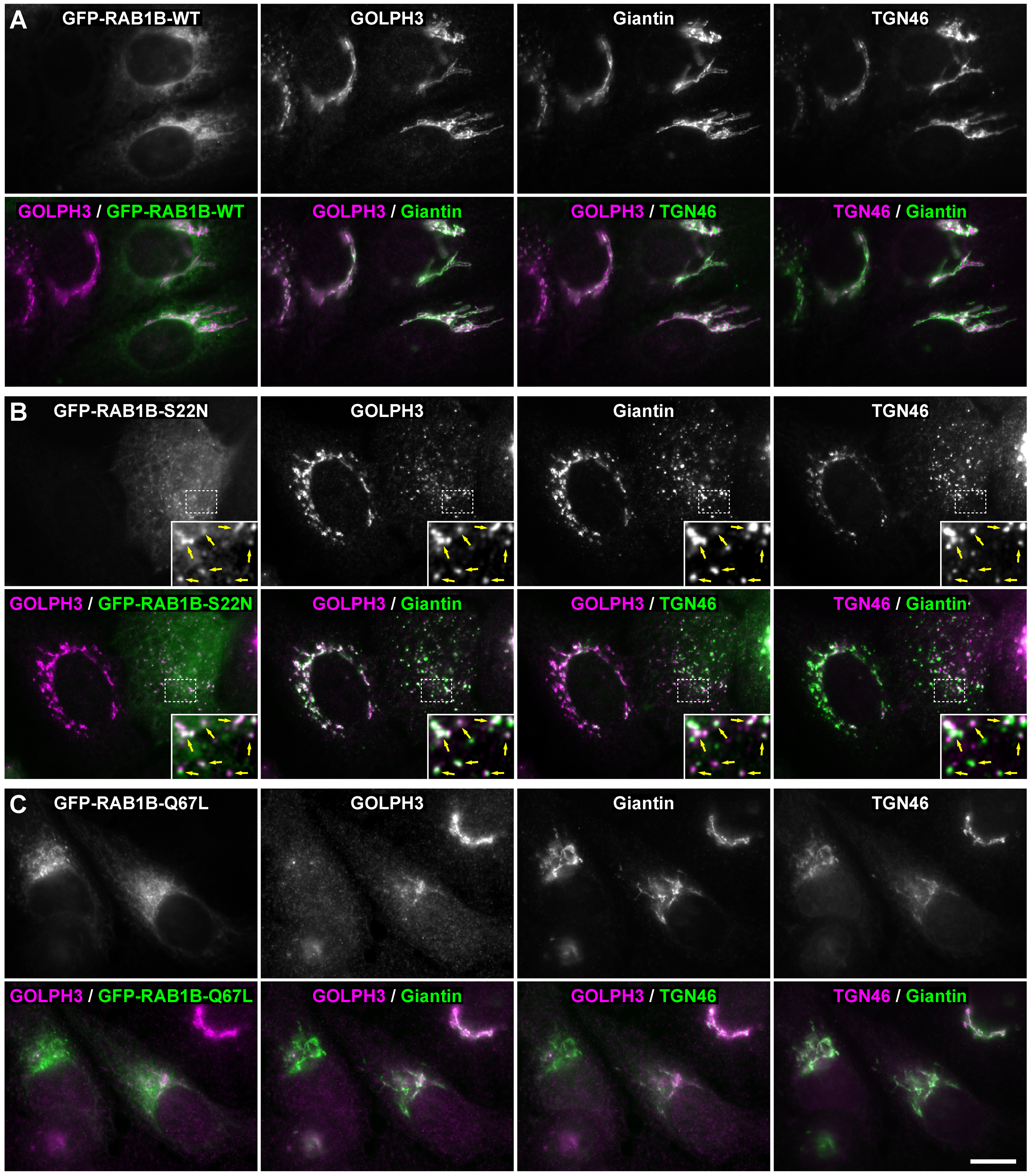

Supplement: S2 Fig — Cells were triple-labeled with rabbit polyclonal antibody to GOLPH3, mouse monoclonal antibody to Giantin and sheep polyclonal antibody to TGN46. Secondary antibodies were Alexa-Fluor-594-conjugated donkey anti-rabbit IgG, Alexa-Fluor-647-conjugated donkey anti-mouse IgG and Alexa-Fluor-350-conjugated donkey anti-sheep IgG. Stained cells were examined by fluorescence microscopy. Insets in B: X3 magnification, with arrows indicating colocalization at Golgi punctae. Bar, 10 μm. For comparison of the fluorescence signals, pairs of images were pseudocolored as indicated. (TIF) [file pone.0237514.s002.tif]

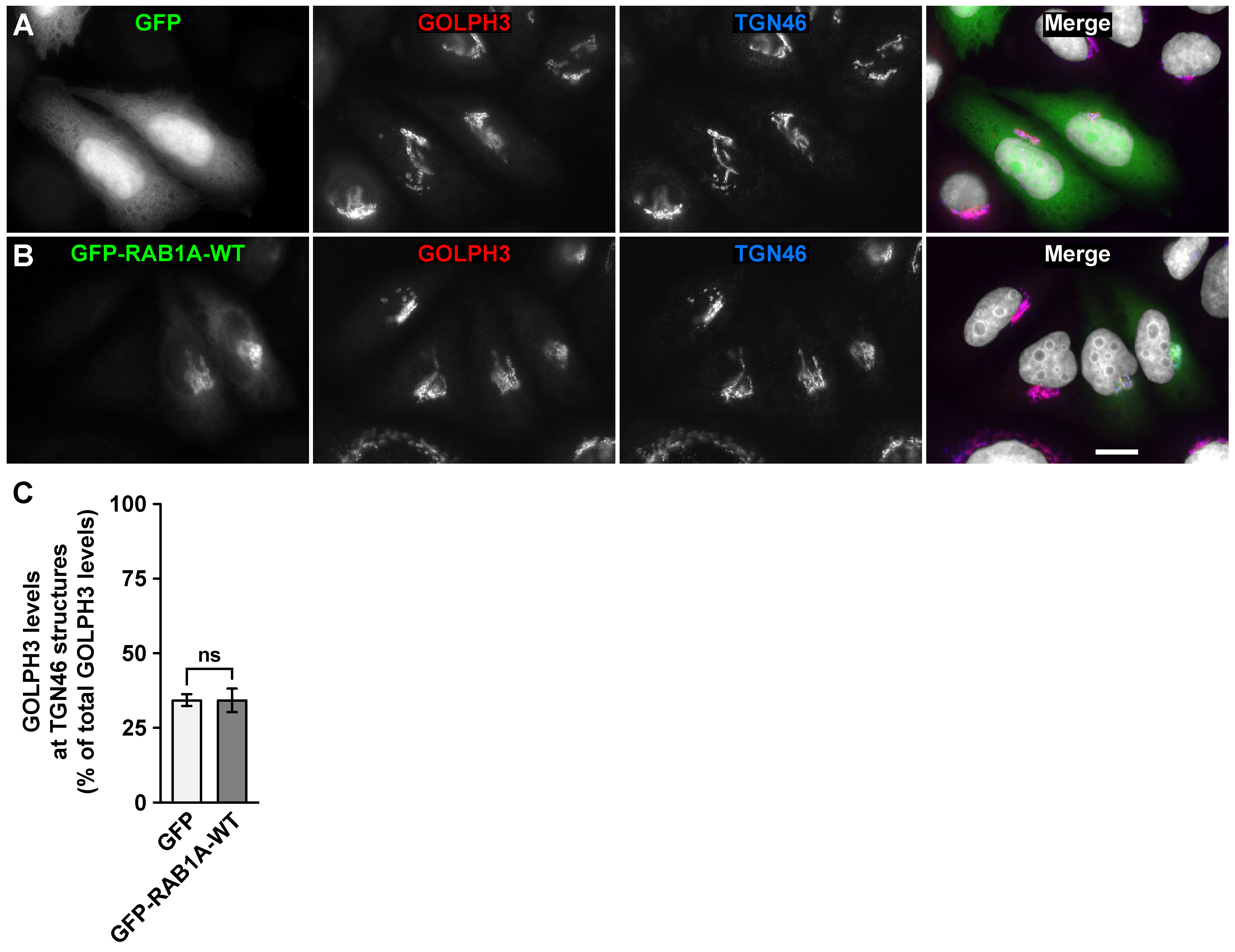

Supplement: S3 Fig — H4 cells grown in glass coverslips were transfected to express GFP (used as control; A, green channel), or the wild type GFP-tagged variant of RAB1A (B, green channel). Cells were fixed, permeabilized, and double-labeled with rabbit polyclonal antibody to GOLPH3 and sheep polyclonal antibody to TGN46. Secondary antibodies were Alexa-594-conjugated donkey anti-rabbit IgG (red channels) and Alexa-647-conjugated donkey anti-sheep IgG (blue channels). Nuclei were stained with DAPI (gray channels). Stained cells were examined by fluorescence microscopy. Merging green, red, blue and grey channels generated the fourth image on each row; yellow indicates overlapping localization of the red and green channels, cyan indicates overlapping localization of the green and blue channels, magenta indicates overlapping localization of the red and blue channels, and white indicates overlapping localization of all three channels. Bar, 10 μm. (C) Quantification as described in Materials and Methods of the percentage of fluorescence signal of anti-GOLPH3 associated to Golgi elements decorated with anti-TGN46. Bar represents the mean ± standard deviation (n = 3 independent experiments, and 15 cells in each experiment were analyzed); ns, not statistically significant. (TIF) [file pone.0237514.s003.tif]

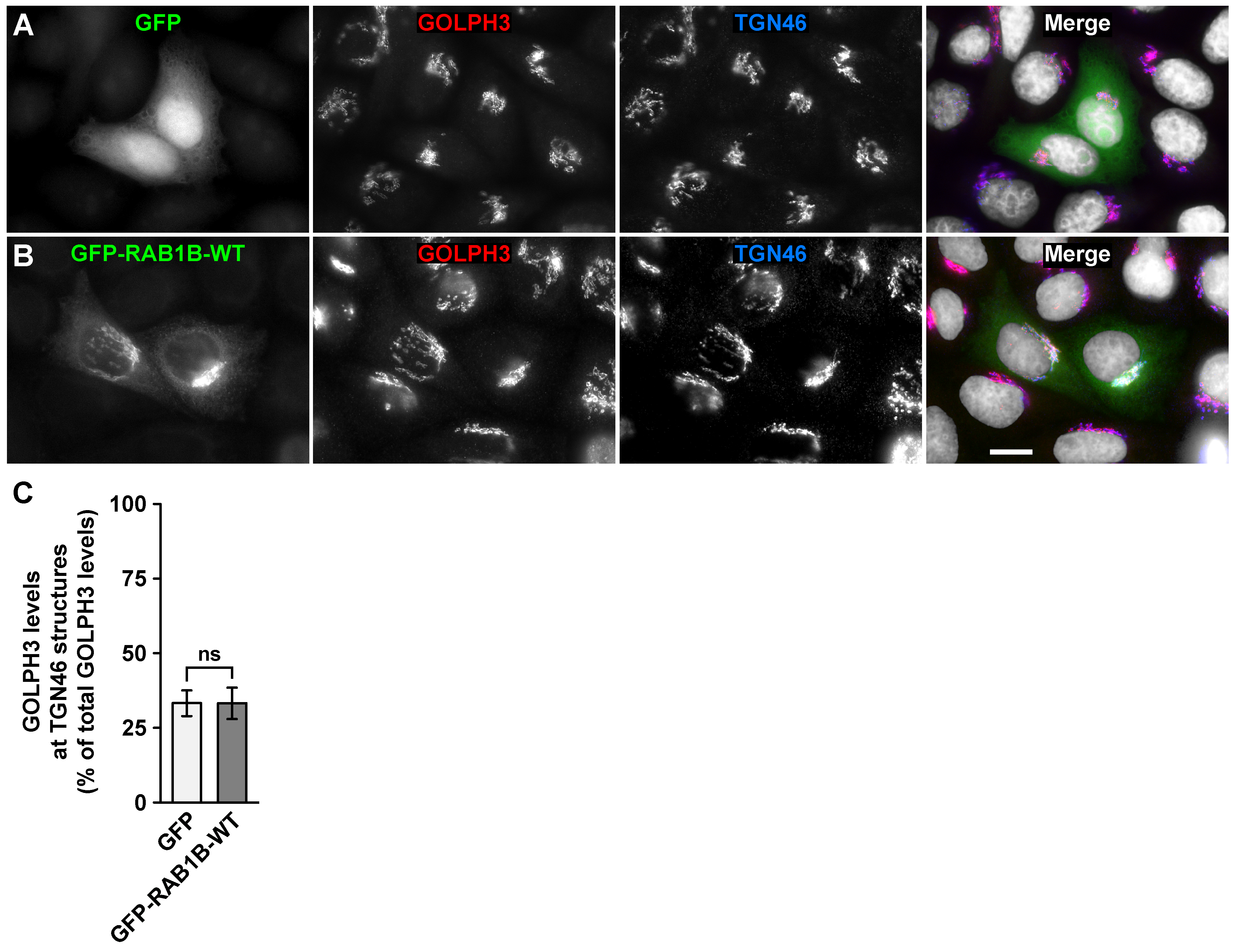

Supplement: S4 Fig — H4 cells grown in glass coverslips were transfected to express GFP (used as control; A, green channel), or the wild type GFP-tagged variant of RAB1B (B, green channel). Cells were fixed, permeabilized, and double-labeled with rabbit polyclonal antibody to GOLPH3 and sheep polyclonal antibody to TGN46. Secondary antibodies were Alexa-594-conjugated donkey anti-rabbit IgG (red channels) and Alexa-647-conjugated donkey anti-sheep IgG (blue channels). Nuclei were stained with DAPI (gray channels). Stained cells were examined by fluorescence microscopy. Merging green, red, blue and grey channels generated the fourth image on each row; yellow indicates overlapping localization of the red and green channels, cyan indicates overlapping localization of the green and blue channels, magenta indicates overlapping localization of the red and blue channels, and white indicates overlapping localization of all three channels. Bar, 10 μm. (C) Quantification as described in Materials and Methods of the percentage of fluorescence signal of anti-GOLPH3 associated to Golgi elements decorated with anti-TGN46. Bar represents the mean ± standard deviation (n = 3 independent experiments, and 15 cells in each experiment were analyzed); ns, not statistically significant. (TIF) [file pone.0237514.s004.tif]

Figure 2A

A

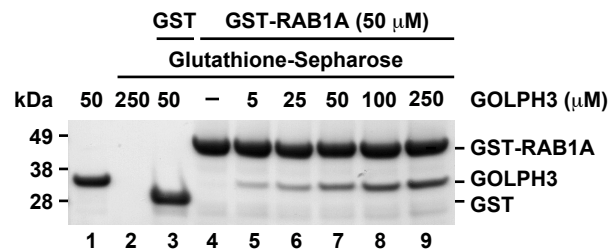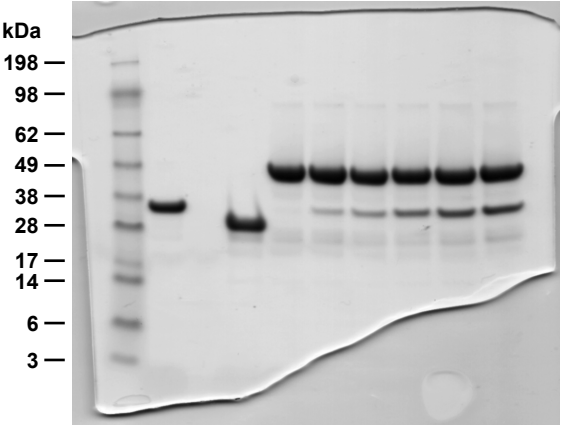

Figure 2C

C

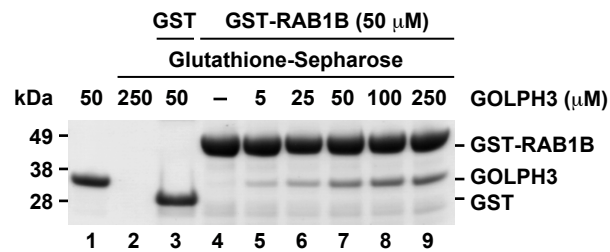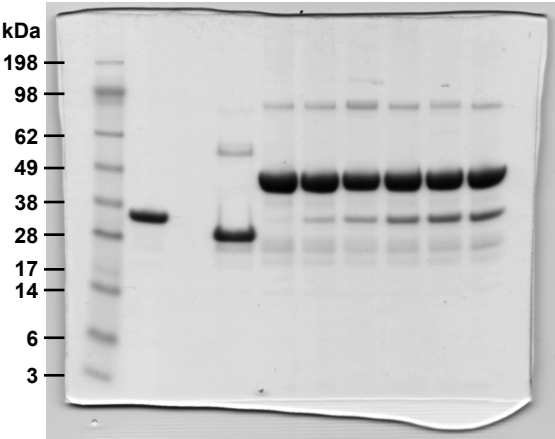

Figure 2E

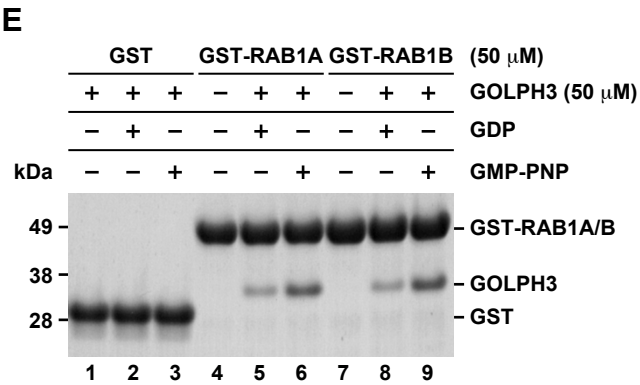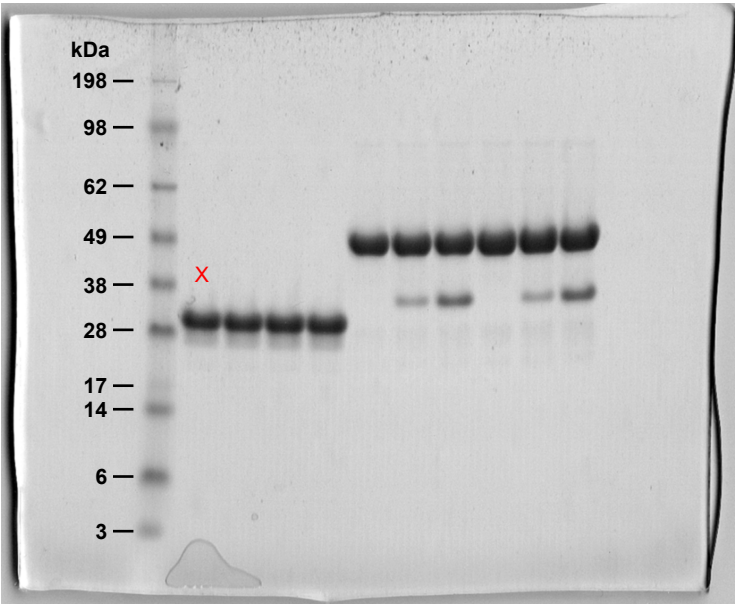

Figure 4A

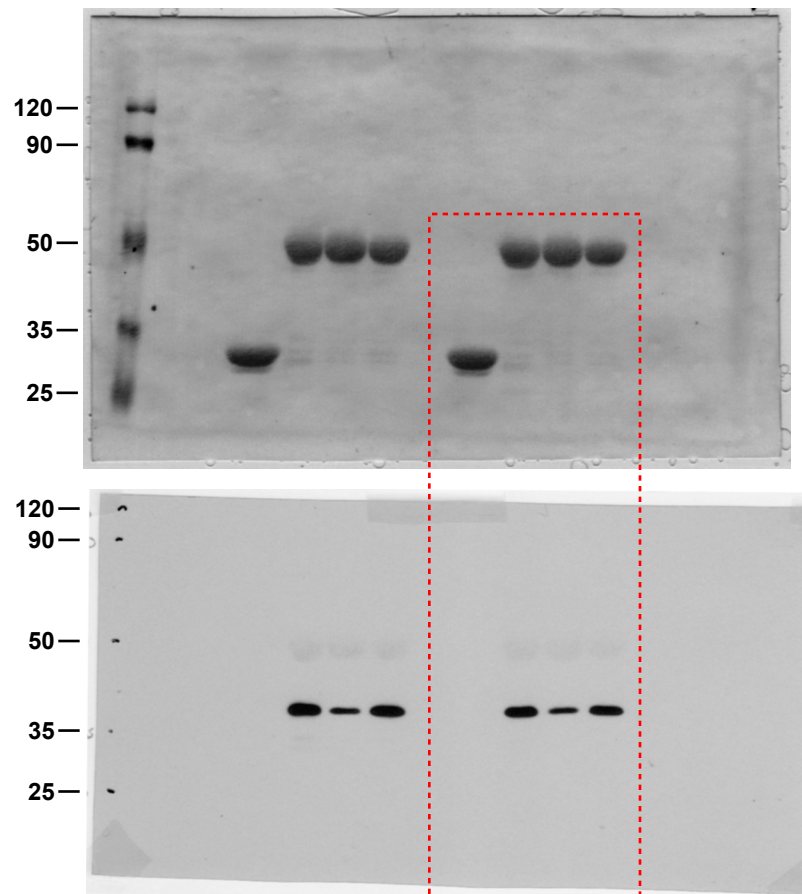

A

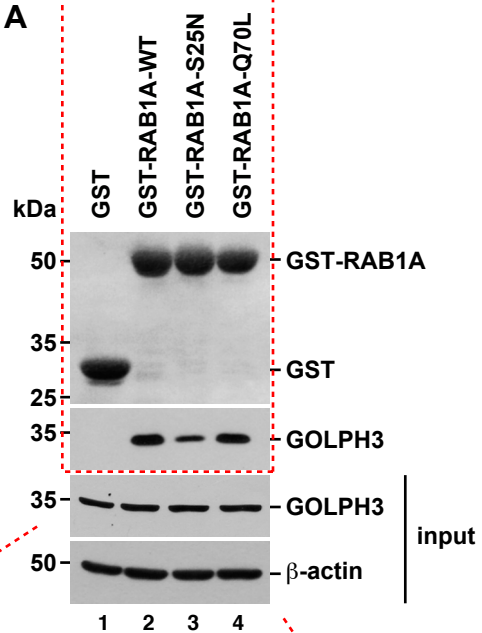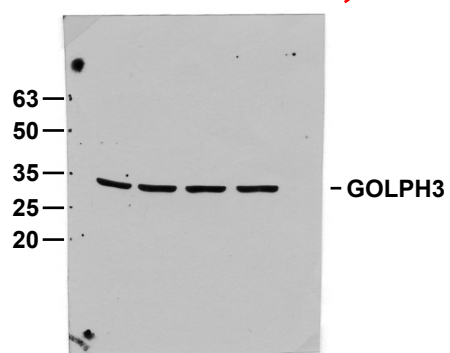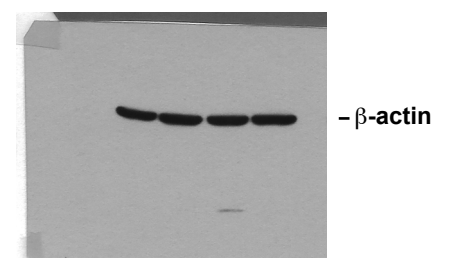

Figure 4B

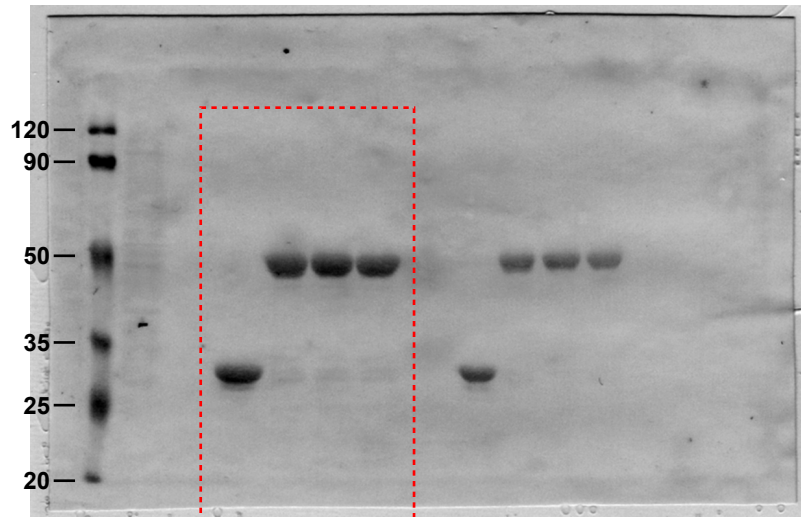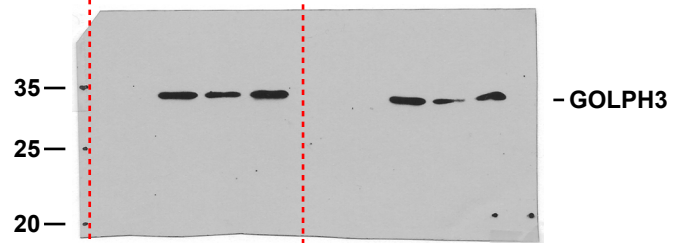

B

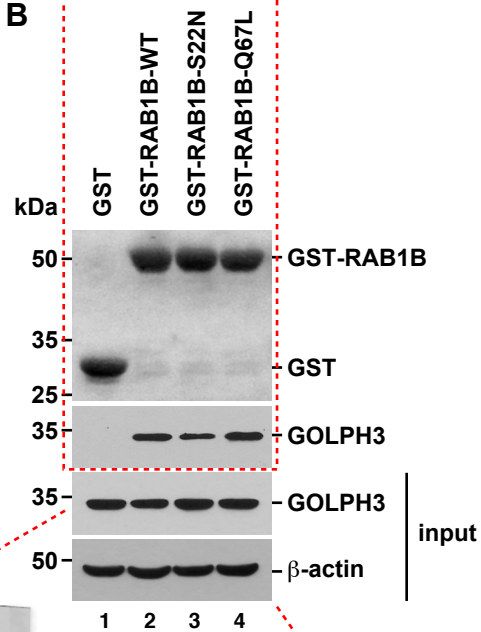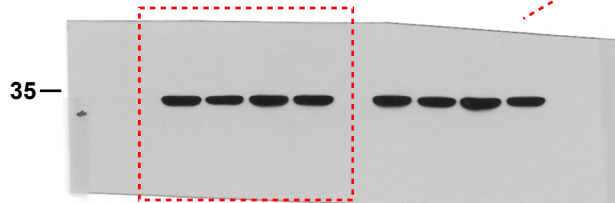

- GOLPH3

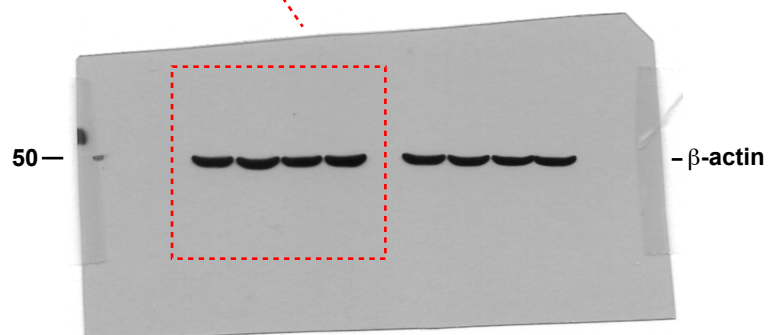

-  $\beta$ -actin

Figure 5A

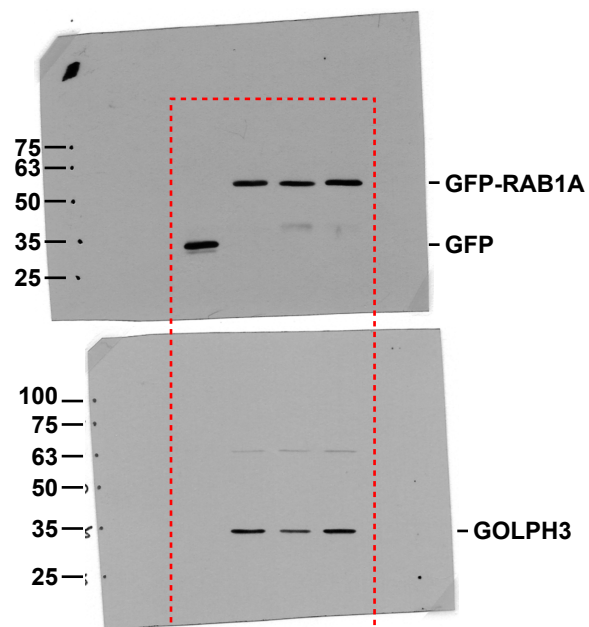

**B**

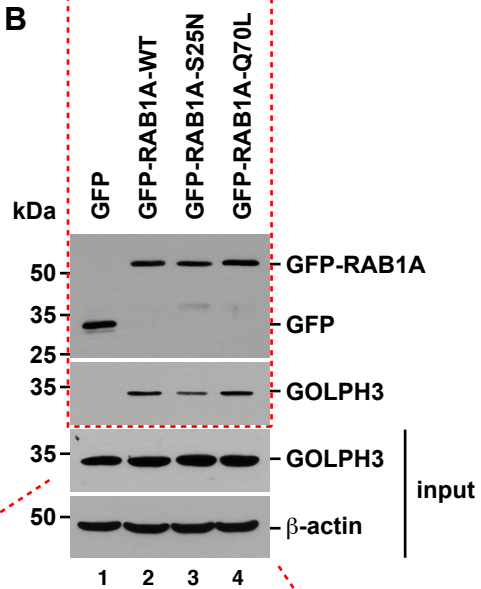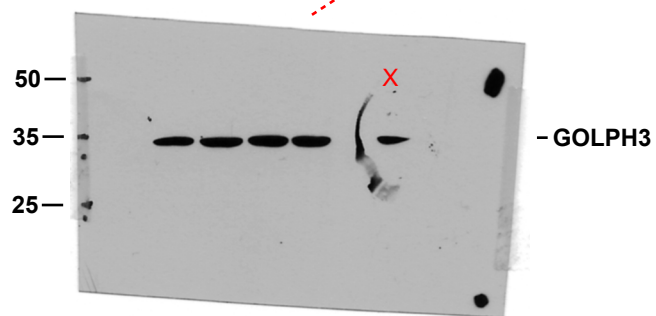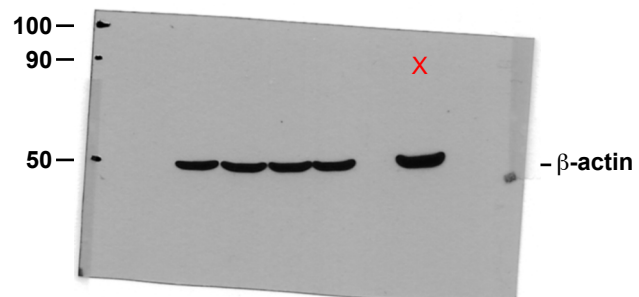

Figure 5B

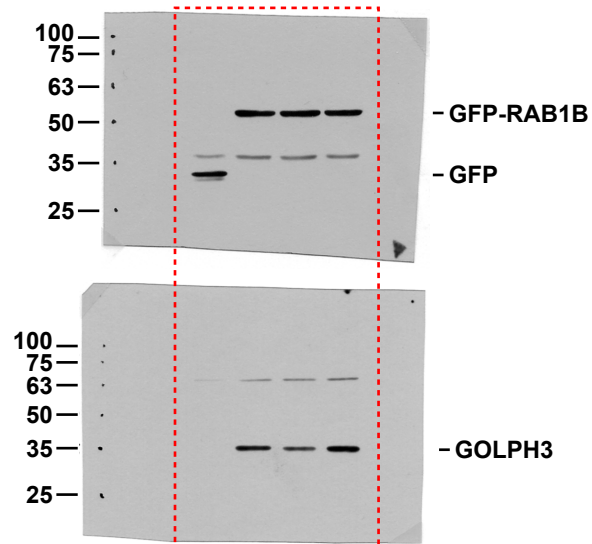

**B**

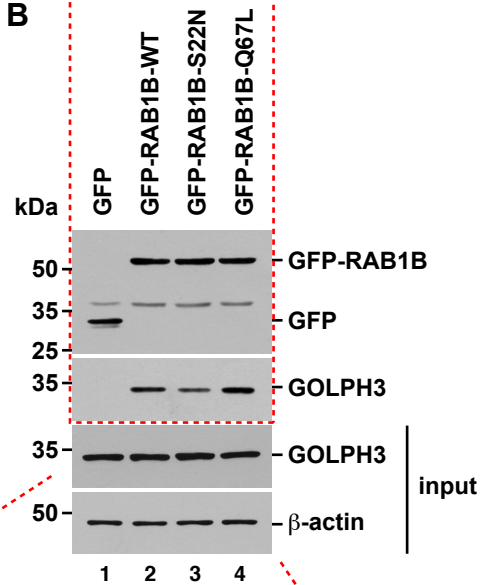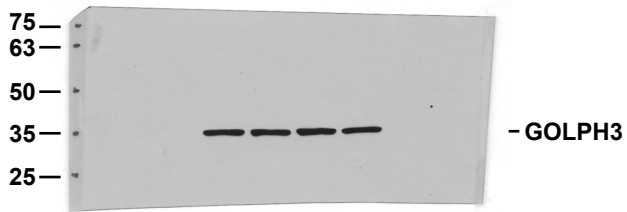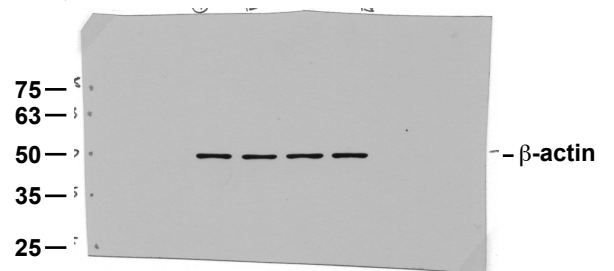

Supplement: S1 Raw images — (PDF) [file pone.0237514.s005.pdf]
